# Supplementary material for: Comparative Visualization of the RNA Suboptimal Conformational Ensemble In Vivo
Source: Biophys J. 2017 Jun 15;113(2):290–301. doi: 10.1016/j.bpj.2017.05.031 (PMC5529173; doi:10.1016/j.bpj.2017.05.031)
Supplement: Document S1. Supporting Materials and Methods, EnsembleRNA software documentation, and Figs. S1–S9 [file mmc1.pdf]

Biophysical Journal, Volume *113*

Supplemental Information

Comparative Visualization of the RNA Suboptimal Conformational Ensemble In Vivo

Chanin T. Woods, Lela Lackey, Benfeard Williams, Nikolay V. Dokholyan, David Gotz, and Alain Laederach

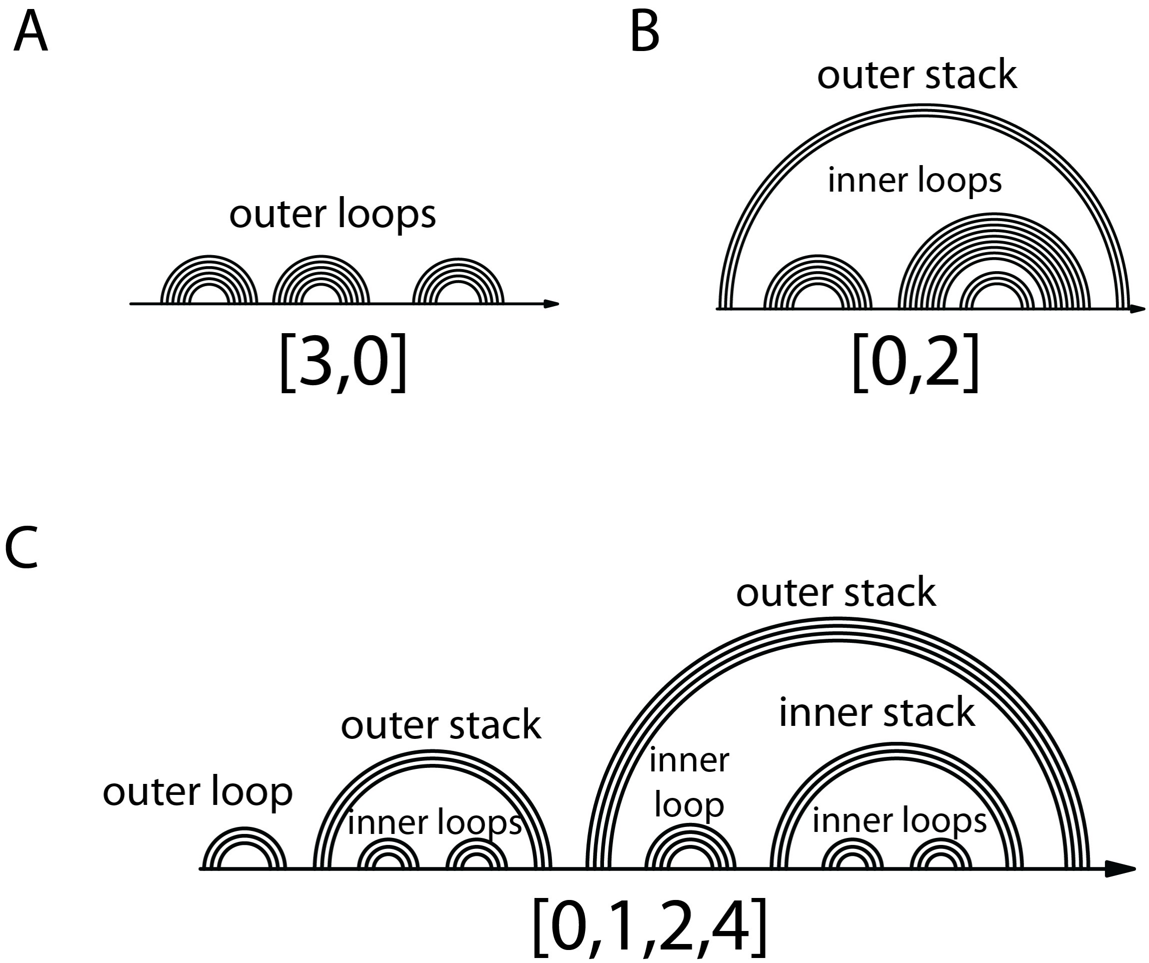

Figure S1. RNA structure abstraction and nestedness. We utilized RNAshapes abstraction to identify unique structure clusters (1). This abstraction assumes that the sizes of structural elements are less important than whether they are present. We used this method to create a numeric vector representation. This representation is based on the nestedness of RNA stacks and loops. A) If only outer loops are present in a structure, we place the number of outer loops in the first column. B) If an outer stack is present, we place the number of loops inside that stack in the n+1 column. C) For each outer loop or outer stack, we place the number of inside loops into the n+1 column.

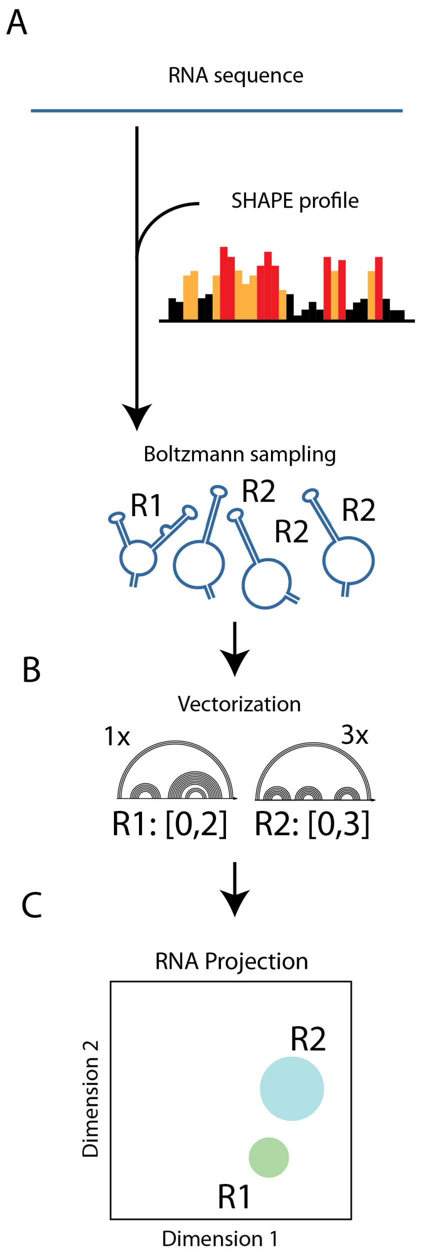

Figure S2. Projection of the reference RNA. A) We generated 1000 structures using Boltzmann-weighted suboptimal sampling from the reference RNA sequence (2). We used experimental structure data collected from selective 2'-hydroxyl acylation analyzed by primer extension (SHAPE) to guide the ensemble prediction (3, 4). Each structure was converted into our nestedness representation (Figure 2E). We retained the orientation of the points from the map of conformational space. The size of each bubble was varied based on the frequency for that structure cluster in the wild type ensemble.

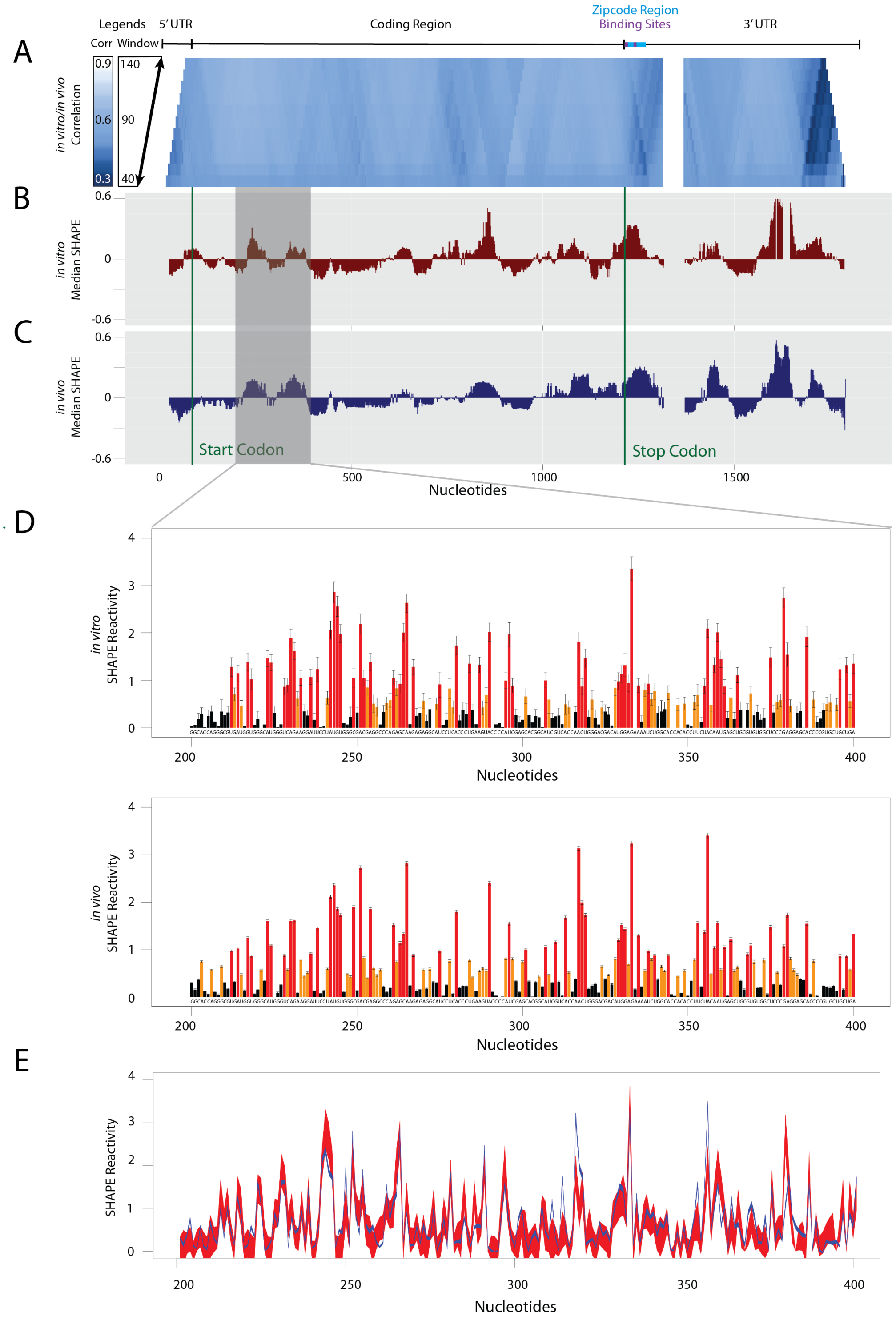

Figure S3. Comparison of similar *in vitro* and *in vivo* structure for the human *β-actin* mRNA. A) We calculated the Pearson correlation in windows between the SHAPE reactivities collected *in vitro* and *in vivo* for the *β-actin* mRNA. For each step of the trapezoid from bottom to top, the window size increases by five nucleotides from 40 to 140. High correlation (white) corresponds to areas that are similar in structure and low correlation (blue) corresponds to areas

that are different in structure. The distances from the median SHAPE value for B) *in vitro* and C) *in vivo β-actin* were calculated in 50 nucleotide windows. Segments with reactivities above the median are less structured than segments with reactivities below the median. The gray panel highlights a region in which the SHAPE reactivity is the same *in vitro* and *in vivo*. D) This similarity is reflected in the *in vitro* (top) and *in vivo* (bottom) SHAPE traces. E) The *in vitro* (red) and *in vivo* (blue) SHAPE traces were overlaid for this region. The thickness of the line corresponds to the error. Structure probing was performed using the high throughput SHAPE-MaP technique. The zipcode region (bright blue) and two zipcode protein-binding sites (purple) are labeled above the windowed correlation and at the bottom of the SHAPE traces.

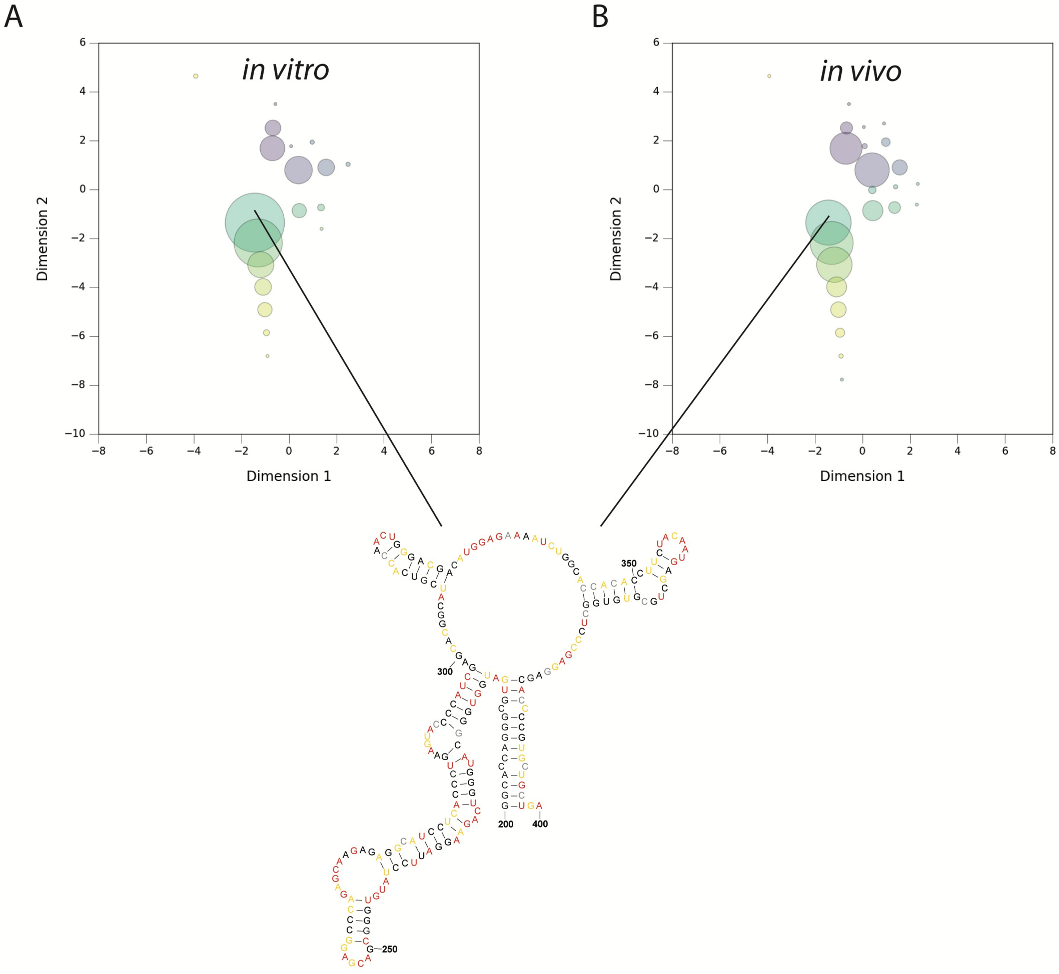

Figure S4. Similar *in vitro* and *in vivo* ensembles for human *β-actin* mRNA. Generation of structures for the *β-actin* mRNA ensemble was guided by the *in vitro* and *in vivo* SHAPE data. The 200-nucleotide regions were folded separately. We compared the visualizations for A) *in vitro* and B) *in vivo* SHAPE-guided ensembles for a region where SHAPE reactivities were expected to be the same. The visualization confirms that the *in vitro* and *in vivo* ensembles are the same. The medoid structure for the most common cluster is shown (center).

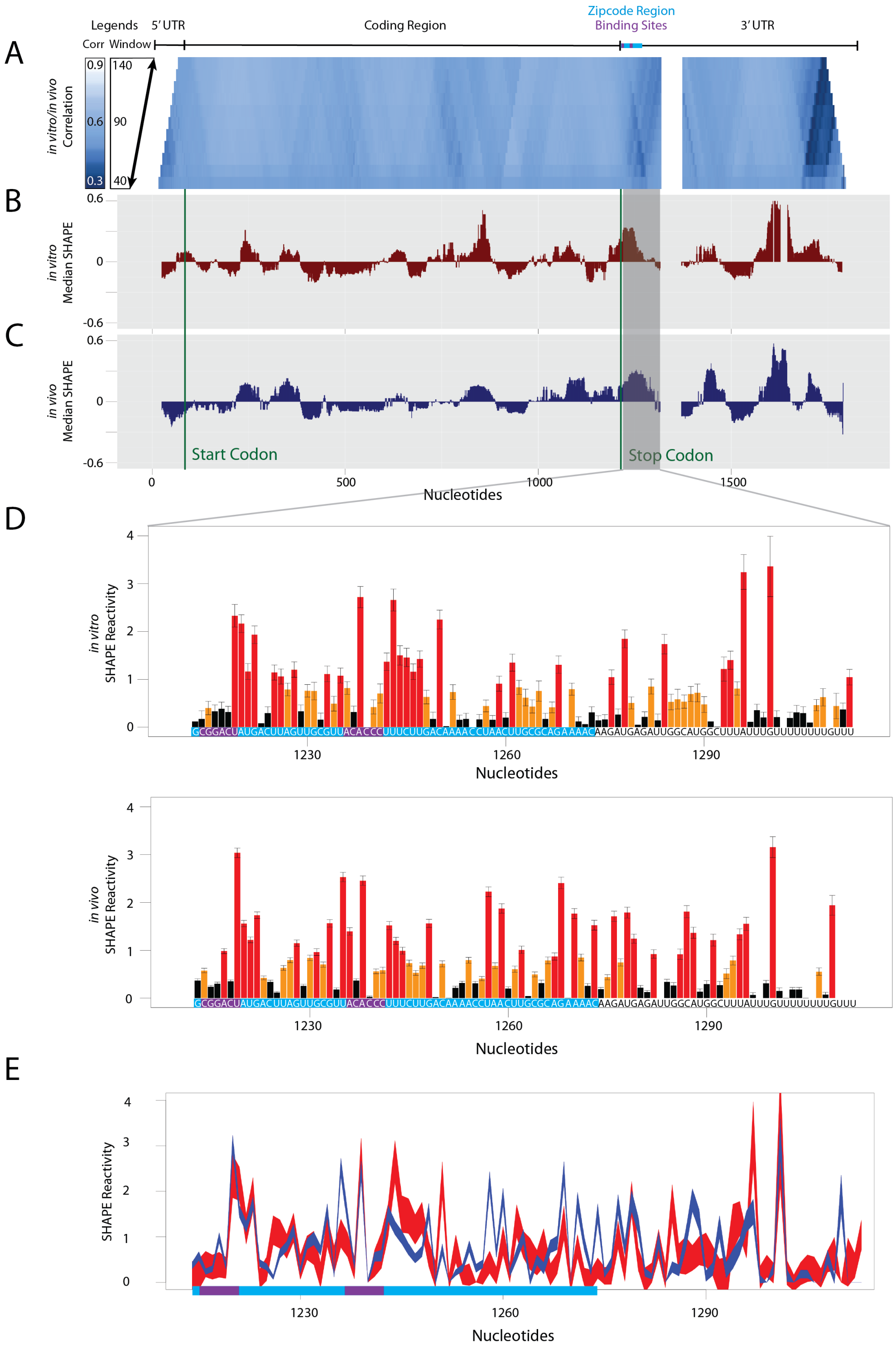

Figure S5. Comparison of different *in vitro* and *in vivo* structure for the human *β-actin* mRNA. A) We calculated the Pearson correlation in windows between the SHAPE reactivities collected *in vitro* and *in vivo* for the *β-actin* mRNA. For each step of the trapezoid from bottom to top, the window size increases by five nucleotides from 40 to 140. High correlation (white)

corresponds to areas that are similar in structure and low correlation (blue) corresponds to areas that are different in structure. The distances from the median SHAPE value for B) *in vitro* and C) *in vivo β-actin* were calculated in 50 nucleotide windows. Segments with reactivities above the median are less structured than segments with reactivities below the median. The gray panel highlights a region in which the SHAPE reactivity is different *in vitro* and *in vivo*. D) This difference is reflected in the *in vitro* (top) and *in vivo* (bottom) SHAPE traces. E) The *in vitro* (red) and *in vivo* (blue) SHAPE traces were overlaid for this region. The thickness of the line corresponds to the error. Structure probing was conducted using the high throughput SHAPE-MaP technique. The zipcode region (bright blue) and two zipcode protein binding sites (purple) are labeled above the windowed correlation and at the bottom of the SHAPE traces.

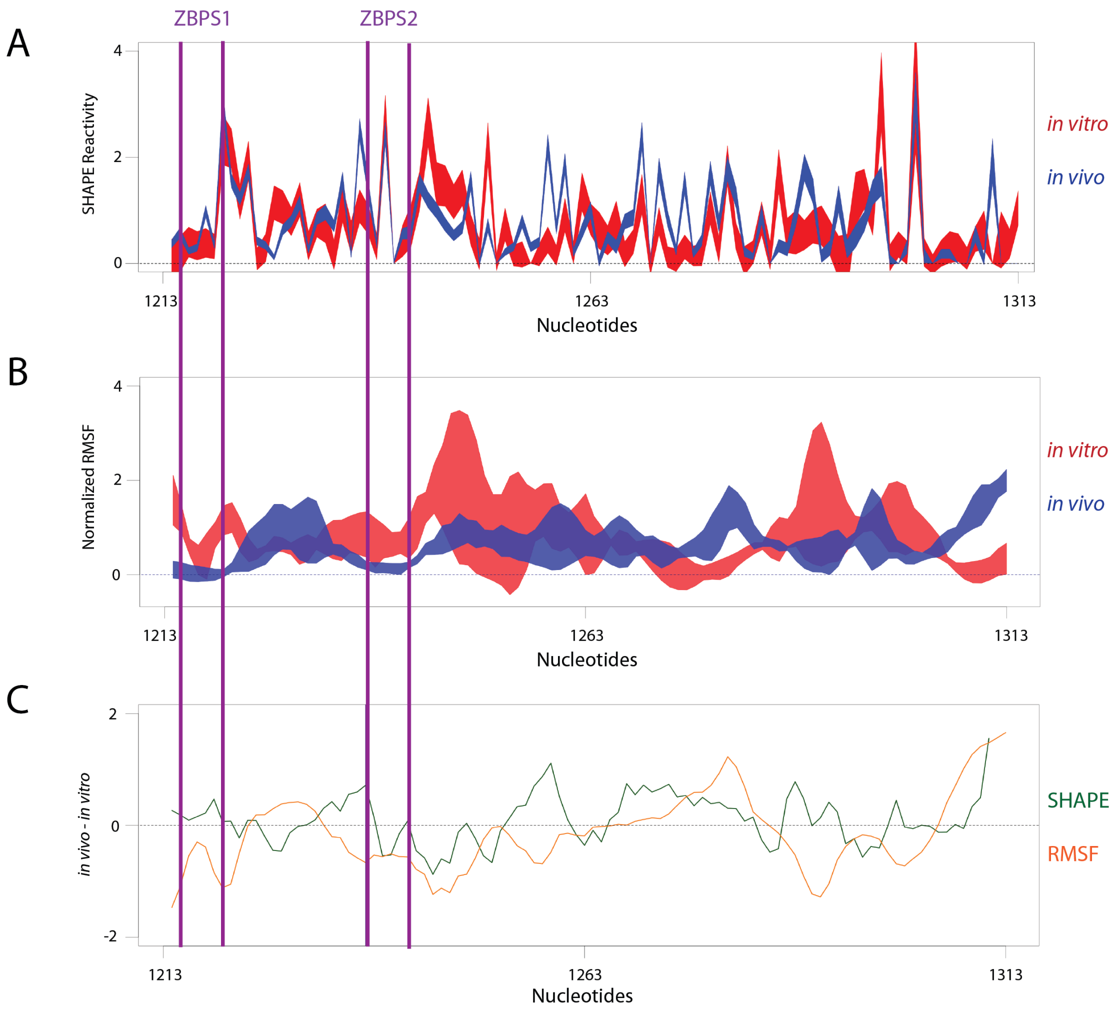

Figure S6. Comparison of different *in vitro* and *in vivo* flexibility for the human *β-actin* mRNA. A) The *in vitro* (red) and *in vivo* (blue) SHAPE traces were overlaid for the zipcode region of *β*-actin. The thickness of the line corresponds to the error. Structure probing was performed using the high throughput SHAPE-MaP technique. B) The normalized Root Mean Square Fluctuation (RMSF) for *in vitro* (red) and *in vivo* (blue) *β*-actin. The fluctuation is calculated from the 3D structural models shown in Figure 5. RMSF values were averaged over a 3-nucleotide moving window. Line thickness corresponds to standard error over three molecular dynamics simulations for each scenario. C) Comparison of the difference between in vivo and in vitro for SHAPE (green) and RMSF (orange). Values above zero indicate higher reactivities or RMSF for the *in vivo* sample. These values were averaged over a 3-nucleotide moving window. Values below zero indicate higher reactivities or RMSF for the *in vitro* sample. The zipcode binding sites are labeled with purple vertical lines for Figures A-C.

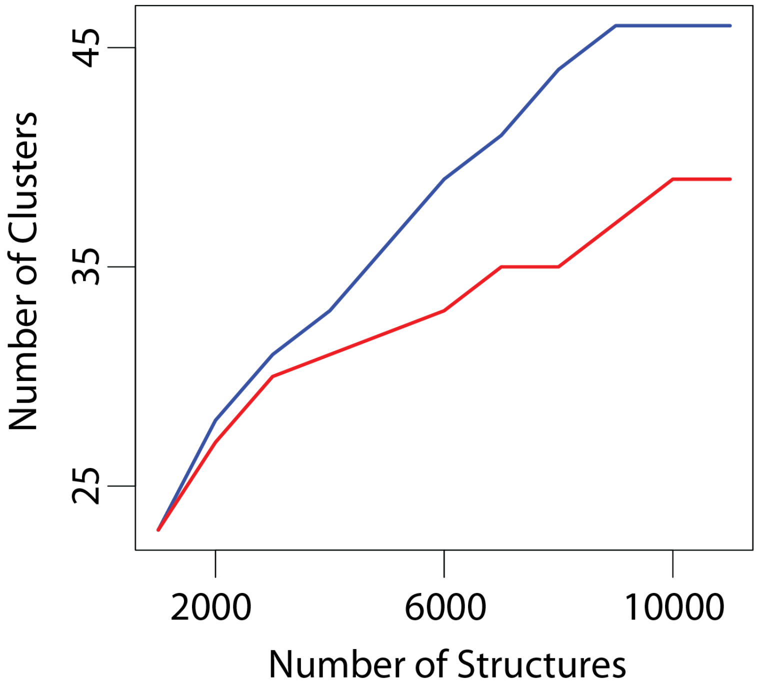

Figure S7. Comparison between single sequence stochastic sampling and single point mutant approach proposed in this manuscript for building a map of conformational space. In this analysis of the 200 nucleotide region of the *β-actin* mRNA shown in Figure S3, we compute the number of clusters as a function of sampled structures for the single point mutant strategy (blue) and single sequence sampling strategies (red). For the same total number of structures, we converge on just 39 structure clusters (red), and 46 using the single point mutant strategy (blue). Thus our strategy generally appears to create a more diverse space that converges on additional structures for ensemble visualization.

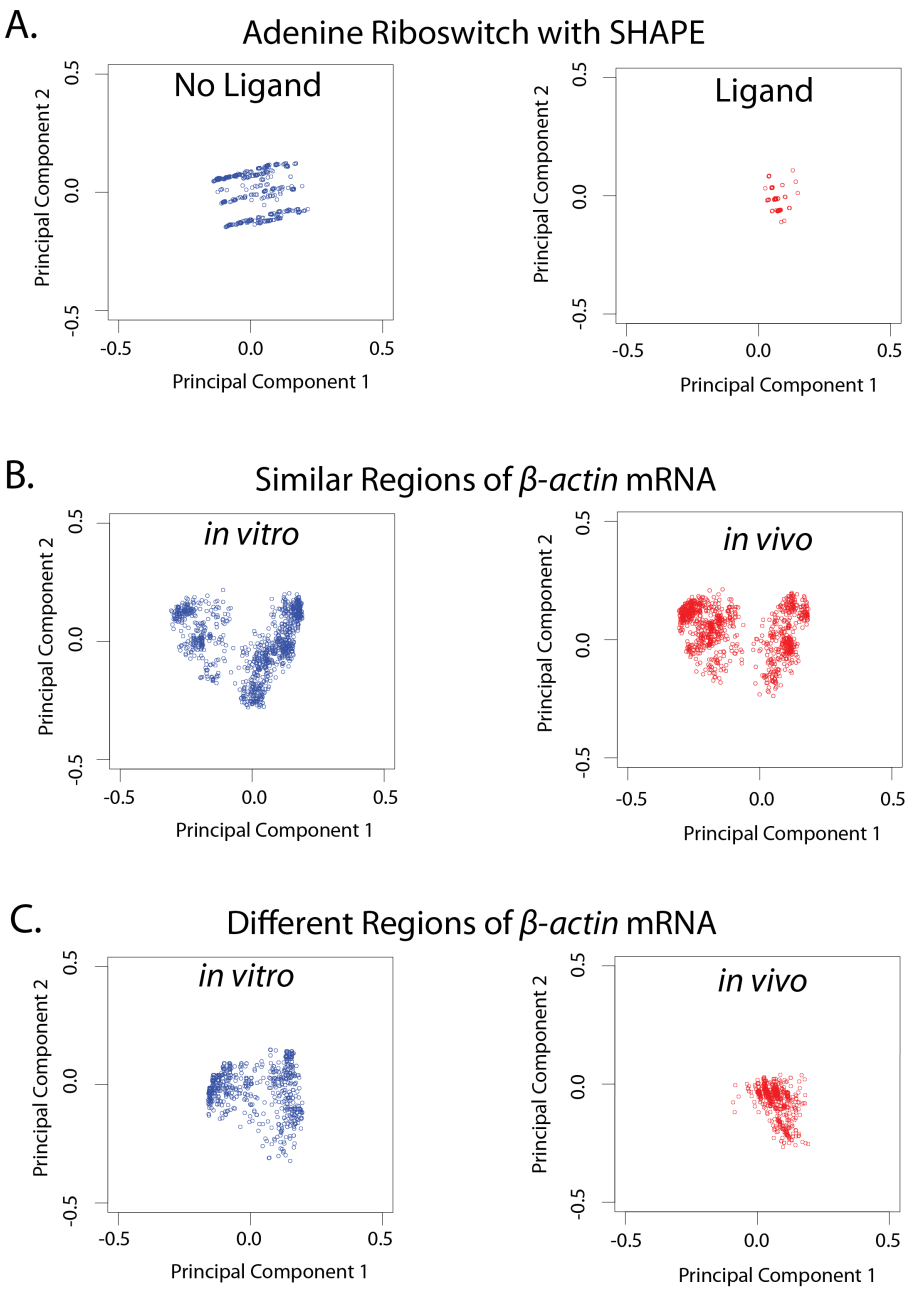

Figure S8. Projection of structural ensembles using principal components analysis (PCA). Representing RNA structure as a binary representation with 0 for unpaired positions and 1 for paired positions, we projected the ensemble onto the first two principal components from PCA (left/blue). Using the same eigenspace, we projected the comparison structural ensemble (right/red). A) We visualize the wild type adenine riboswitch ensemble with SHAPE from Figure 3B. Although the ensembles look different, the shift from "off" conformations to "on" conformations is not obvious in this visualization. B) We projected the region of the *β-actin* mRNA from Figure 4 where we expected the ensembles to be different *in vitro* and *in vivo* based on the Pearson correlation coefficients on the SHAPE-MaP data. The *in vivo* ensemble appears to

be shifting away from the preferred structure *in* vitro, but it is difficult to see the magnitude of the shift based on this visualization. C) Using PCA, we visualized the region of the *β-actin* mRNA from Figure S3 where we expect the ensembles to be the same. The visualization captures the similarity for these ensembles. However, it is difficult to compare the diversity of the ensembles in this region (more diversity of structures) with the ensemble from the region shown in C (less diversity of structures).

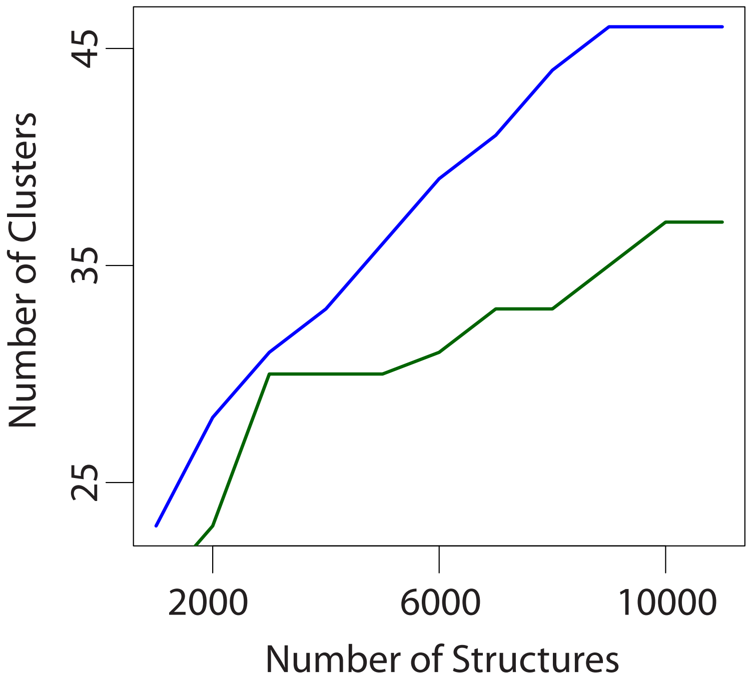

Figure S9. Comparison between hierarchical clustering with Euclidean distance as proposed in this manuscript (blue) and Jenson Shannon divergence (green) for building a map of conformational space. In this analysis of the 200 nucleotide region of the *β-actin* mRNA shown in Figure S3, we compute the number of clusters as a function of the number of mutants chosen from hierarchical clustering with 1000 structures for each mutant. The distances used in hierarchical clustering were the Euclidean distance (blue) and Jensen Shannon divergence (green). For the same total number of mutants, we converge on just 37 structure clusters (green), and 46 using the Euclidean distance strategy (blue). Thus our strategy generally appears to create a more diverse space that converges on additional structures for ensemble visualization that is the goal in this implementation of the visualization. Nonetheless, if users desire a simpler space for visualization, altering the distance metric used at this stage of the approach could be an interesting approach.

Documentation S1.

EnsembleRNA

September 20, 2016

**Title**	Visualize the structural ensemble for a given RNA
**Version**	1.0.0
**Author**	Chanin Tolson Woods
**URL**	http://ribosnitch-ensemblerna.rhcloud.com
**URL2**	http://ribosnitch.bio.unc.edu/software

Description

EnsembleRNA is a package for the visualization and comparison of RNA structural ensembles. This package creates a stable map of conformational space for a given RNA and its mutants. The map explores the most diverse conformational space and generates the structures using established Boltzmann-weighted suboptimal sampling algorithms. Using vector representation based on arc diagram nested loop patterns, EnsembleRNA projects clusters of structures from the map into two dimensions using metric multidimensional scaling. Individual RNA ensembles are visualized in this space by varying the size of the structure clusters in a bubble plot.

The sequence from the FASTA file is the reference used to create the map of conformational space, unless otherwise specified. To compare two reference structures, the same sequence or set of structures must be used to create the map of conformational space. Larger RNAs may require more sequences for a stable visualization. Selective 2'-hydroxyl acylation and primer extension (SHAPE) data can be included to guide the prediction of the reference ensemble.

Note: EnsembleRNA is written for use on a Linux/Unix-type operating system. Use of EnsembleRNA in any instance requires the installation of the numpy, jinja2, ipython, mpld3, matplotlib, scipy, and sklearn modules for Python. Also required is the RNAstructure package from the Mathews lab.

**Depends**	Python 2.7 or Python 3.5
**License**	GPL (>=3)
**Imports**	numpy, jinja2, ipython, mpld3, matplotlib, and sklearn
**Required**	RNAstructure

Table of Contents

Imports and Requirements 3

Installation 3

Usage 4

Options 4

Output 5

Troubleshooting 5

Diagonal line visualization 5

Selecting medoid structure 6

Single point visualization 5

Outer loop visualization 7

Increasing map coverage 8

Documentation References 9

Imports and Requirements

For use on a Linux/Unix operating system

1) python (recommended version 2.7 or 3.5)

2) numpy (recommended 1.11.0)
   pip install numpy

3) scipy (recommended version 0.17.1)
   pip install scipy

4) sklearn (recommended version 0.0)
   pip install sklearn

5) jinja2 (recommended version 2.8)
   pip install jinja2

6) ipython (recommended version 4.2.0)
   pip install ipython

7) mpld3 (recommended version 0.2)
   pip install mpld3

8) matplotlib (recommended version 1.5.1)
   pip install matplotlib

9) RNAstructure (recommended version 5.8)
   http://rna.urmc.rochester.edu/RNAstructureWeb/
   Download command-line applications for your platform
   Extract to /usr/local/bin (or directory of your choice)
   Add following 2 lines to ~/.bash_profile (path may be different)
      export PATH=$PATH:/usr/local/bin/RNAstructure/exe
      export DATAPATH=/usr/local/bin/RNAstructure/data_tables

Installation

1) Download requirements listed above

2) Download EnsembleRNA package

3) Place package in /usr/local/bin (or directory of your choice)

4) tar -zxvf ensemblerna (extract)

5) cd ensemblerna (enter extracted directory)

6) sudo python setup.py install (install ensemblerna as python module)

7) ensemblerna -h (test installation in any directory)

Usage

ensemblerna <fasta file> <output directory> [options]

Options

General

-h, --help	show this help message and exit
-v, --version	show program's version number and exit

Inputs

-sh --shape	Includes shape data in the reference ensemble prediction. Ignored if -d flag is used (Default is None)
-d --db	Dot-bracket structures for reference ensemble (Default is None)
-m --map	Sequence to create the map of conformational space. Ignored if -md flag is used (Default is reference fasta file)
-md --mapdb	Dot-bracket structures for the map of conformational space. A previously created map can be used to project new ensembles onto the same space (Default is None)
-s --size	Number of mutants for the map of conformational space. Higher numbers increase structural diversity. Ignored if –md flag is used (Default is 10)
-p --plotmap	Plot the map T/F (Default is T)
-r --range	Range of nucleotides to visualize. Predicted structures will include the full length of the input RNA but only the given range will be plotted (Default is 1 to sequence length)
-pi --plotinteractive	Plot the interactive file T/F (Default is T)
-th --threadmax	Maximum number of threads for multi-threading. (Default is 1)
-i --ignorestems	Ignore stems with fewer than i base pairs. (Default is 3)
-n --num	Number of Boltzmann sampled structures to produce for the visualization (Default is 1000)

RNAstructure

-maxd --maxdistance	The maximum number of bases between the two nucleotides in a pair (Default is no restriction)
-t --temperature	Temperature at which the calculation takes place in Kelvin (Default is 310.15 K)
-si --SHAPEintercept	The intercept used with SHAPE restraints. Ignored if -d flag is used (Default is -0.6 kcal/mol)
-sm --SHAPEslope	The slope used with SHAPE restraints. Ignored if -d flag is used. (Default is 1.8 kcal/mol)

Output

For both reference and map of conformational space
.csv CSV file with cluster number, cluster size, and representative structure
.db Dot-Bracket file with structures
.pdf PDF file with visualization plot
.png PNG file with visualization plot

Interactive visualization
.html HTML file with interactive plotting

Troubleshooting

Diagonal line visualization

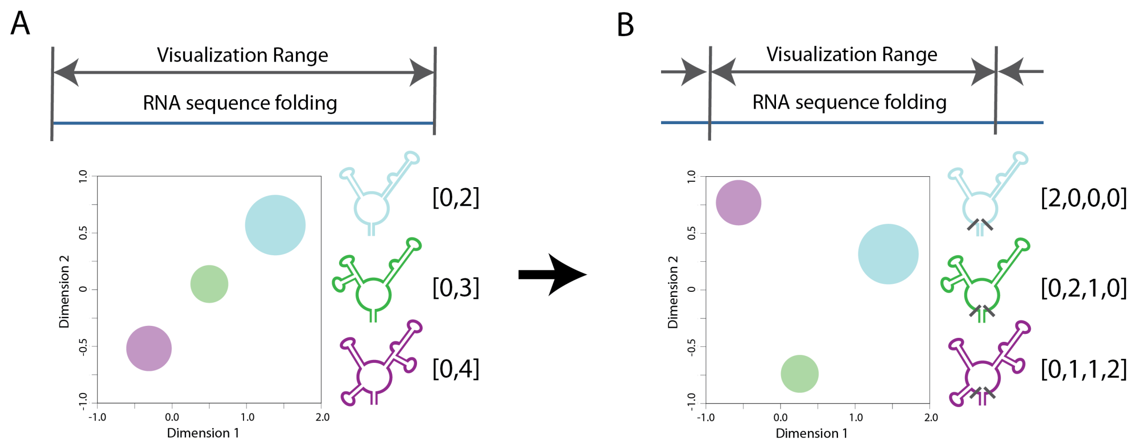

Problem If all bubbles lie on a diagonal line, there is correlation between dimensions 1 and 2 (A). By default, EnsembleRNA defines RNA structure based on the outermost stacks and loops (the most abstracted representation). In this case, the structures are similar from the level of the outermost stack, but interesting differences may exist for loops within that outer stack.

Solution To address this problem, the full-length of the RNA can be folded, while the visualization is focused on a shorter range that excludes the outer stack (B). This change reveals the more subtle differences between structures.

Example For a 250 nucleotide RNA, include the entire sequence in the fasta file. Only visualize the range from nucleotide 50 to 200 using the range flag (-r or --range).

ensemblerna <fasta file> <output directory> **-r 50 200**

Selecting medoid structure

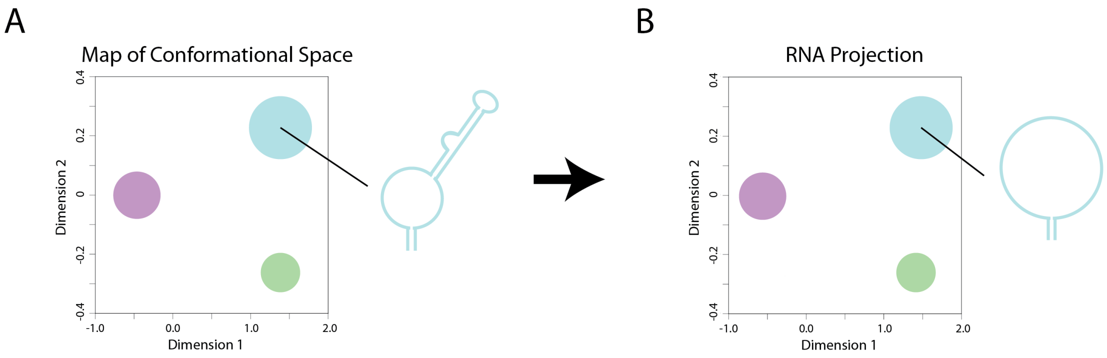

Problem The default medoid structure is chosen from the map of conformational space (A). Using this medoid keeps the representation consistent between different ensembles projected onto the same space. However, the best representative structure for the projected RNA may be different.

Solution A structure selected from the projected RNA ensemble may be more representative (B). Alternatively, the minimum free energy structure from either the map or the projected RNA ensemble can be used.

Example Check the .db file in the output folder.

Single point visualization

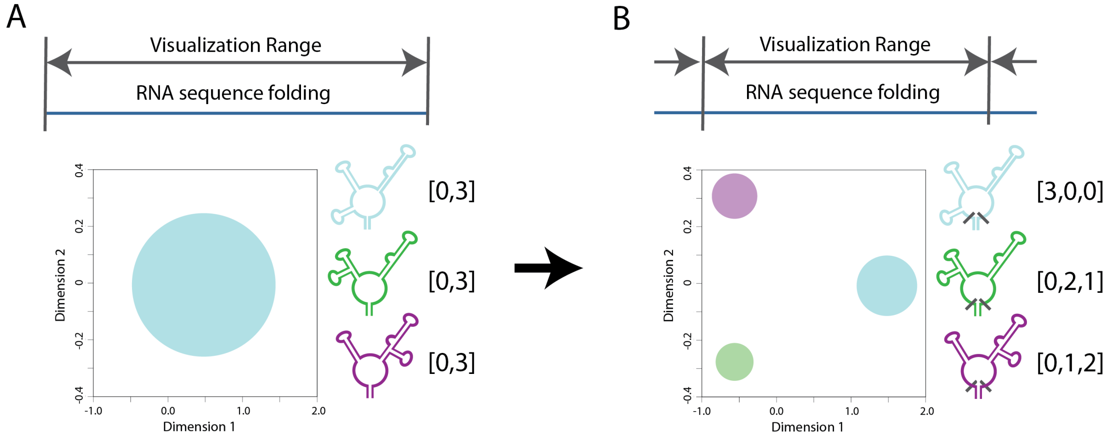

Problem If all structures are placed in a single cluster, the visualization may be too broad (A). By default, EnsembleRNA defines RNA structure based on the outermost stacks and loops (the most abstracted representation). In this case, the structures are the exact same from the level of the outermost stack, but interesting differences may exist for loops within that outer stack.

Solution To address this problem, the full-length of the RNA can be folded, while the visualization is focused on a shorter range that excludes the outer stack (B). This change reveals the more subtle differences between structures.

Example For a 250 nucleotide RNA, include the entire sequence in the fasta file. Only visualize the range from nucleotide 50 to 200 using the range flag (-r or --range).

ensemblerna <fasta file> <output directory> **-r 50 200**

Outer loop visualization

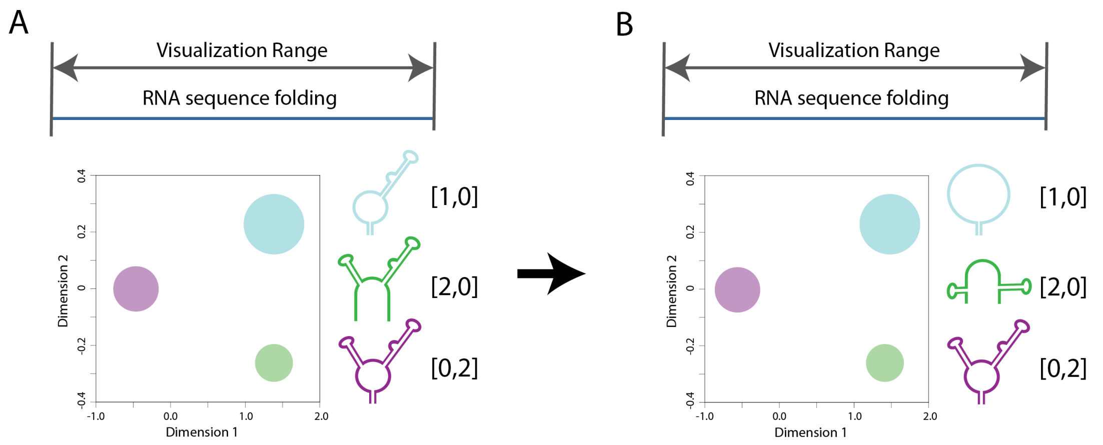

Problem RNA clusters with only outer loops are considered to be more similar to each other than those clusters with an outer stack (A). The clusters with only outer loops are most often very diverse groupings. While some structures may be similar to those with outer stacks, many structures will be quite different.

Solution Our nestedness representation method accounts for this increased diversity in clusters with only outer loops (B). Looking at the cluster medoid may be useful in assessing the similarity of these clusters to those with outer stacks.

Example Check the .csv file or the .html file in the output folder.

Increasing map coverage

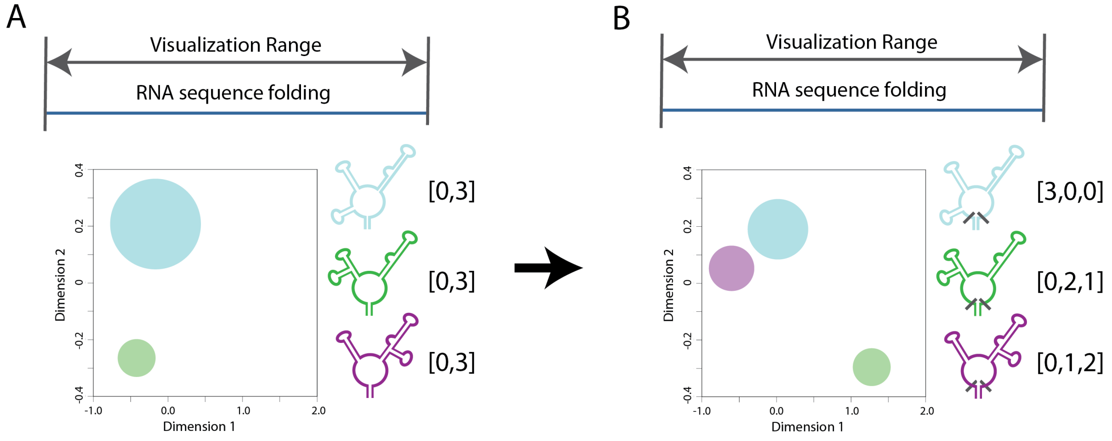

Problem The default map size (the number of single point mutants included in the map) is automatically set to 10. This is a reasonably size for RNAs of shorter length (100-200 nucleotides). However, longer RNAs will likely require larger map sizes to sufficiently explore the structural space for an RNA.

Solution Increase the map size in increments until the number of clusters structures converges. At this map size, additional single point mutants will not increase the structural diversity in the map of conformational space. The optimal map size varies by RNA.

Example For an 800 nucleotide RNA, include the entire sequence in the fasta file. Increase the map size from 10 to 100.

ensemblerna <fasta file> <output directory> **-s 100**

Documentation References

Suboptimally sampled structures are generated using the RNAstructure package
http://rna.urmc.rochester.edu/RNAstructureWeb/
Version 5.8 (references 2, 3, 4, and 5)

1. D.H. Mathews. 2004. Using an RNA Secondary Structure Partition Function to Determine Confidence in Base Pairs Predicted by Free Energy Minimization. RNA, 10:1178-1190. (2004).
2. S. Duan, D.H. Mathews, D. H. Turner. 2006. Interpreting Oligonucleotide Microarray Data to Determine RNA Secondary Structure. Application to the 3' End of Bombyx mori R2 DNA. Biochemistry, 45:9819-9832.
3. D.H. Mathews. 2006. Revolutions in RNA Secondary Structure Prediction. Journal of Molecular Biology, 359:526-532.
4. S. Wuchty, W. Fontana, I. L. Hofacker, P. Schuster. 1999. Complete Suboptimal Folding of RNA and the Stability of Secondary Structures. Biopolymers, 49:145-165.
5. Y. Ding and C.E. Lawrence. 2003. A Statistical Sampling Algorithm for RNA Secondary Structure Prediction. Acids Research, 31:7280-7301.
6. E.J. Merino, K. A. Wilkinson, J. L. Coughlan and K. M. Weeks. 2005. RNA structure analysis at single nucleotide resolution by selective 2-hydroxyl acylation and primer extension (shape). J Am Chem Soc, 127:4223-4231.
7. K.E. Deigan, T. W. Li, D. H. Mathews and K. M. Weeks. 2009. Accurate SHAPE-directed RNA Structure Determination. Proceedings of the National Academy of Sciences USA, 106:97-102.
8. K. Pearson. 1901. On Lines and Planes of Closest Fit to Systems of Points in Space. Philosophical Magazine, 11:559–572.
9. D.B. Carr, R. J. Littlefield, W.L. Nicholson, J.S. Littlefield. 1987. Scatterplot Matrix Techniques for large N. Journal of the American Statistical Association, 389:424-436.
10. J. Ritz, J. Martin and A. Laederach. 2012. Evaluating our ability to predict the structural disruption of RNA by SNPs. BMC Genomics, 13(Suppl 4):S6.

Supporting References

1. P. Steffan, V. Bjorn, M. Rehmsmeier, J. Reeder and R. Giegerich. 2006. RNAshapes: an integrated RNA analysis package based on abstract shapes. 22:500-503.
2. Y. Ding and C. Lawrence. 2003. A statistical sampling algorithm for RNA secondary structure prediction. 31:7280-7301.
3. D. H. Matthews, M. D. Disney, J. L. Childs, S. J. Schroeder, M. Zuker and D. H. Turner. 2004. Incorporating Chemical Modification Constraints into a Dynamic Programming Algorithm for Prediction of RNA Secondary Structure. 101:7287-7293.
4. N. A. Siegfried, S. Busan, G. M. Rice, J. A. Nelson and K. M. Weeks. 2014. RNA motif discovery by SHAPE and mutational profiling (SHAPE-MaP). 11:959-965.
