# Supplementary material for: Comparative Visualization of the RNA Suboptimal Conformational Ensemble In Vivo
Source: Biophys J. 2017 Jun 15;113(2):290–301. doi: 10.1016/j.bpj.2017.05.031 (PMC5529173; doi:10.1016/j.bpj.2017.05.031)
Supplement: Document S2. Article plus Supporting Material [file mmc3.pdf]

Biophysical Journal
Article

Comparative Visualization of the RNA Suboptimal Conformational Ensemble In Vivo

Chanin T. Woods,[1,2] Lela Lackey,[2] Benfeard Williams,[3] Nikolay V. Dokholyan,[3] David Gotz,[4,5] and Alain Laederach[1,2,*]

[1]Curriculum in Bioinformatics and Computational Biology, [2]Department of Biology, [3]Department of Biochemistry and Biophysics, [4]Carolina Health Informatics Program, and [5]School of Information and Library Science, University of North Carolina at Chapel Hill, Chapel Hill, North Carolina

ABSTRACT When a ribonucleic acid (RNA) molecule folds, it often does not adopt a single, well-defined conformation. The folding energy landscape of an RNA is highly dependent on its nucleotide sequence and molecular environment. Cellular molecules sometimes alter the energy landscape, thereby changing the ensemble of likely low-energy conformations. The effects of these energy landscape changes on the conformational ensemble are particularly challenging to visualize for large RNAs. We have created a robust approach for visualizing the conformational ensemble of RNAs that is well suited for in vitro versus in vivo comparisons. Our method creates a stable map of conformational space for a given RNA sequence. We first identify single point mutations in the RNA that maximally sample suboptimal conformational space based on the ensemble's partition function. Then, we cluster these diverse ensembles to identify the most diverse partition functions for Boltzmann stochastic sampling. By using, to our knowledge, a novel nestedness distance metric, we iteratively add mutant suboptimal ensembles to converge on a stable 2D map of conformational space. We then compute the selective 2′ hydroxyl acylation by primer extension (SHAPE)-directed ensemble for the RNA folding under different conditions, and we project these ensembles on the map to visualize. To validate our approach, we established a conformational map of the *Vibrio vulnificus add* adenine riboswitch that reveals five classes of structures. In the presence of adenine, projection of the SHAPE-directed sampling correctly identified the on-conformation; without the ligand, only off-conformations were visualized. We also collected the whole-transcript in vitro and in vivo SHAPE-MaP for human *β-actin* messenger RNA that revealed similar global folds in both conditions. Nonetheless, a comparison of in vitro and in vivo data revealed that specific regions exhibited significantly different SHAPE-MaP profiles indicative of structural rearrangements, including rearrangement consistent with binding of the zipcode protein in a region distal to the stop codon.

INTRODUCTION

Ribonucleic acid (RNA) 3D structures are the result of remarkably complex interaction networks that together create emergent biological functions (1–4). Although crystal structures reveal these networks with atomic detail, these remain static snapshot models of the conformations existing in the cellular environment (5). RNAs, particularly highly structured RNAs such as ribosomal RNA, exist in multiple conformations, many of which are likely to affect their function(s) (6–8). Thus, when describing RNA structure, it is more accurate to discuss an ensemble of conformations instead of a single structure (7,9–11). However, significant biophysical challenges remain, whether at the secondary or tertiary structural level, including visualization of the ensemble of RNA conformations and identification of essential functional elements within the entire ensemble (9,12–14).

The challenge of visualizing an RNA secondary structure ensemble is easily illustrated by the *Vibrio vulnificus* adenosine deaminase (*add*) adenine riboswitch (Fig. 1) (15–18). Typically RNA is represented as a single structure, but, for the riboswitch, at least two structures are required for function: the on-conformation and the off-conformation (Fig. 1 *A*) (16,18,19). These two structures interchange, with the off-conformation favored without the adenine ligand, and the on-conformation stabilized by binding adenine (17,18,20). Thus, in solution the RNA exists as an ensemble of conformations that interchange (1,8,10,21–23). In visualizing such an ensemble, two salient aspects should be highlighted to understand function: 1) the structural similarity and difference between the two conformations and 2) the relative abundance of each conformation in the ensemble.

Submitted February 14, 2017, and accepted for publication May 19, 2017.
*Correspondence: alain@unc.edu
Editor: Tamar Schlick.
http://dx.doi.org/10.1016/j.bpj.2017.05.031
© 2017 Biophysical Society.
This is an open access article under the CC BY license (http://creativecommons.org/licenses/by/4.0/).

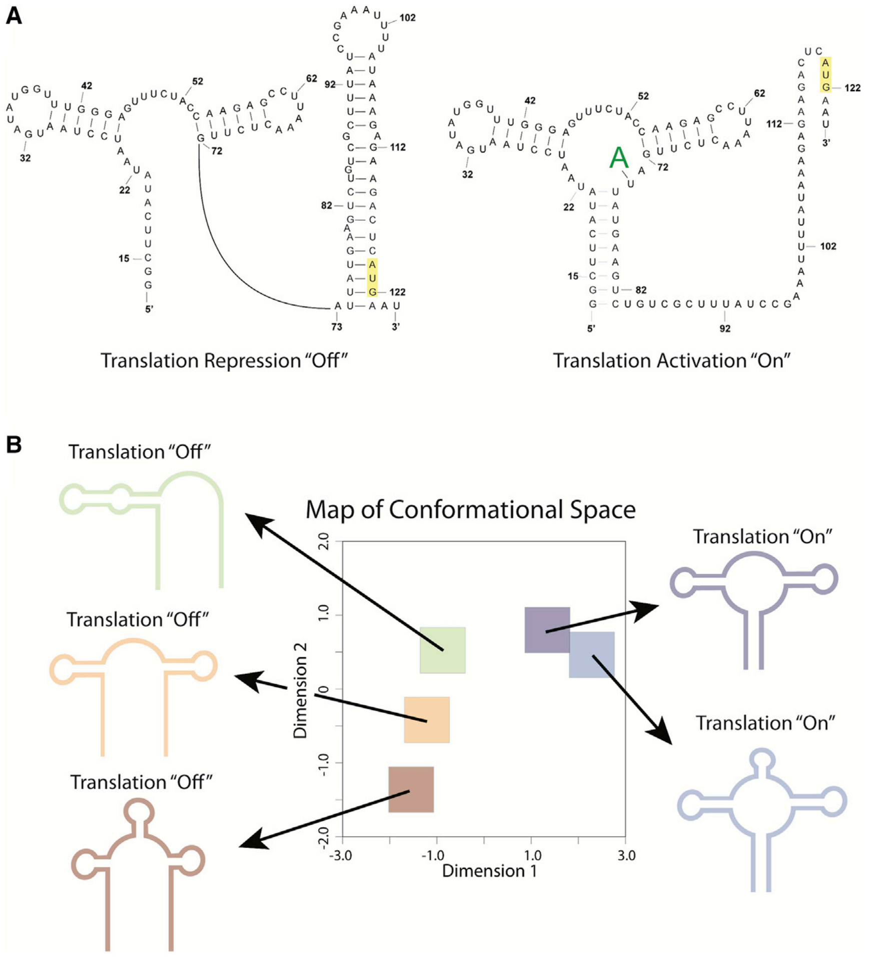

FIGURE 1 The conformational states of the *V. vulnificus add* adenine riboswitch. (*A*) The accepted structures for the bound and unbound states of the riboswitch are determined by crystallography and NMR (54). The unbound state represses translation, and the bound state activates translation (20,54). (*B*) The map of conformational space explores five possible structure clusters for the riboswitch. The representative structure is the cluster medoid structure. The orange cluster represents the translation off-conformation, and the purple cluster represents the translation on-conformation, as confirmed by crystallography and NMR (90). To see this figure in color, go online.

Defining structural similarity requires a representation that captures biologically important structural features of the RNA to facilitate clustering of highly similar conformations. From these clusters, it is then possible to determine the relative abundance of the conformations, which reflects their relative thermodynamic weights in the Boltzmann ensemble. We therefore aim to create a visualization based on a sampling of conformational space like the one illustrated for the *add* riboswitch (Fig. 1 *B*), which was stochastically sampled from the Boltzmann ensemble. In Fig. 1 *B*, we illustrate a map of conformational space, in which each square represents a cluster of similar conformations based on a nested feature vector that we define below. This representation is particularly interesting as it reveals several aspects of the *add* riboswitch conformational ensemble that are not apparent when considering only two structures (Fig. 1 *A*). First, this visualization suggests that there are more than two classes of conformations in the *add* riboswitch conformational ensemble. Second, the on- and off-conformational change is conveniently captured along dimension 1. The methods we describe below provide a robust approach for identifying specific dimensions that capture biologically informative structural differences, such as those in Fig. 1 *B*.

In Fig. 1 *B*, we purposely did not indicate the relative abundance of conformations in each conformational cluster; each square is equal in size. The relative weight of these clusters depends on the underlying thermodynamic parameters of the energy model. Given a nearest-neighbor energy model, it is now computationally efficient to rapidly sample the Boltzmann suboptimal ensemble (24–27). Furthermore, the nearest neighbor model can be extended to empirically include experimental structure probing data, particularly selective 2′ hydroxyl acylation by primer extension (SHAPE) data (28,29). Inclusion of SHAPE data is relevant because the RNA structure is readily probed under different experimental conditions. For example, the *add* riboswitch can be probed with and without the ligand that causes a structural rearrangement (15,30,31). As we will show below, the visualization proposed in Fig. 1 *B* accurately captures this biologically important rearrangement when combined with SHAPE-informed structure probing.

Although visualizing riboswitch ensemble conformations is one important goal of our work, the main motivation for improving the ability to visualize and interpret RNA conformational ensembles stems from our studies of messenger RNA (mRNA) folding in vitro versus in vivo. Quantitative

comparison of these two conditions effectively enables us to deconvolute the effect of the cellular environment on mRNA folding. The structural ensembles of these long and flexible RNAs tend to be far more complex than the structural ensembles of riboswitches. As such, we require tools that enable "sorting the forest from the trees" to understand these large and complex molecules. We present here an experimental high-resolution comparison of SHAPE data for the human *β-actin* mRNA that reveals specific regions in which the RNA folds differently in vitro versus in vivo. We show how these visualizations enable interpretation of the complex rearrangements of the mRNA conformational ensemble that occur in the cell, thereby obtaining meaningful biophysical and biological insight into the specific structure function relationships of the specific messenger. Together, these novel data and methods, to our knowledge, establish a robust approach for interpreting chemical and enzymatic probing data in the context of conformational ensembles.

MATERIALS AND METHODS

Generating structures for the map of conformational space

Our strategy for establishing a conformational map of an RNA ensemble is illustrated in Fig. 2. Beginning with the RNA sequence (Fig. 2 *A*), we compute its partition function (probability of basepairing (32–35)) and the partition functions of all AtoU, UtoA, CtoG, and GtoC single point mutant sequences (Fig. 2 *B*). These point mutations are experimentally determined to be maximally disruptive of structure (32). The purpose of stochastic sampling of multiple single point mutant sequences is to generate a more diverse ensemble of structures from which to build a visualization space. This strategy converges faster and generates a more diverse ensemble than traditional stochastic sampling of a single sequence, as can be seen in Fig. S7. The sum over the rows in the partition function is the basepairing probability, *P*, for each nucleotide with every other nucleotide, x_{ij} (Eq. 1). Our goal is to generate an ensemble of diverse possible conformations and establish a representative 2D map for visualization. Thus, single point mutants with the highest ensemble Shannon entropy (*H*), as defined by Eq. 1, are selected for further analysis. This definition of Shannon entropy has previously been used to interpret RNA structure (36–38), and computes the entropy based on the 1D basepairing probability vector. Alternative definitions of Shannon entropy could potentially be used to compute the Shannon entropy from the full 2D basepairing probability matrix, the thermodynamic structural entropy, or algorithm computation (33,34,37). In the first pass, we eliminate the lowest 25% Shannon entropy mutants (Fig. 2 *C*) (36–38). In a second filter, we perform hierarchical clustering of the basepairing probability $P(x_i)$ vectors based on their Euclidean distance (39) to identify the most divergent partition functions (Fig. 2 *D*). We then perform Boltzmann stochastic sampling on the two most divergent partition functions (Fig. 2 *E*), and create nestedness feature vectors from the sampled structures (Fig. 2 *F*; Fig. S1), to generate a map of conformational space using metric multidimensional scaling (40) (Fig. 2 *H*). We iteratively add additional Boltzmann ensemble samples of divergent single point mutant sequences until the map of conformational space converges (Fig. 2 *G*), as follows:

$$H_i = -\sum_{j=1}^{J} P(x_{i,j}) \log_{10} P(x_{i,j}). \quad (1)$$

Projection of the map of conformational space

Our projection is based on the representation proposed in the RNAshapes abstraction that captures whether a stem or stack element exists, ignoring

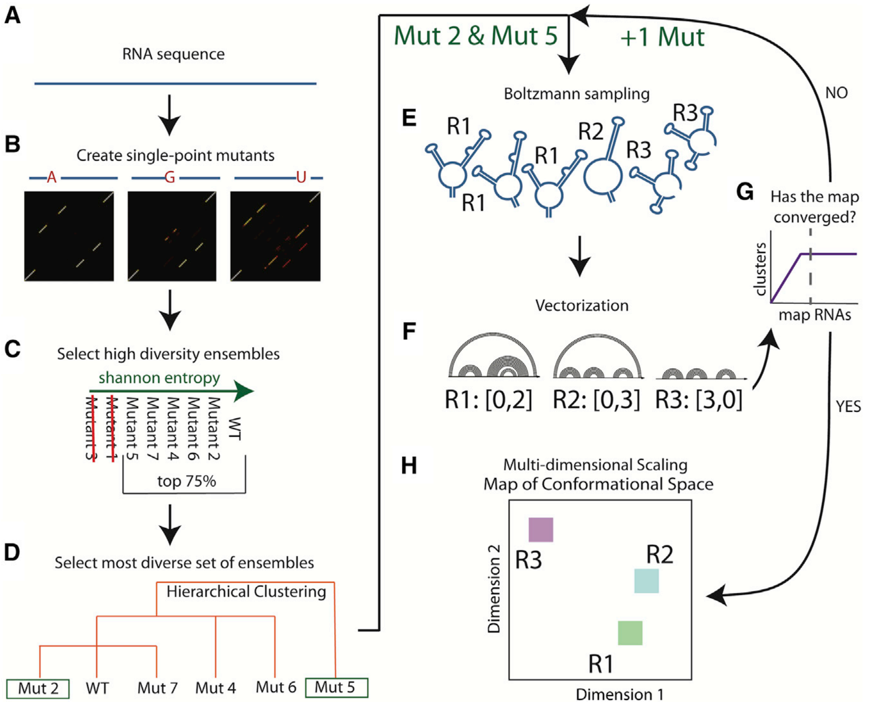

FIGURE 2 Building the map of conformational space. The map explores the possible structural space for an RNA sequence and its single point mutants. (*A*) A single point mutant was created for every position in the RNA. We used only mutations that were expected to lead to the largest changes in structure based on experimental observations from the mutate-and-map experiments (AtoU, UtoA, CtoG, and GtoC) (32,91). (*B*) The partition function was generated for the wild-type and single point mutants using established structure prediction methods (22,49,50,92). (*C*) The RNAs were ranked by Shannon entropy, and the top 75% were retained to filter for individual RNAs with more diverse ensembles (36–38). (*D*) We collapsed the partition function for each of the remaining RNAs into their basepairing probabilities, and performed hierarchical clustering on the probabilities (39). This clustering selects the most diverse RNA subsets. (*E*) We selected the most distant RNA and sampled 1000 structures according to their Boltzmann probability (5). (*F*) We used data abstraction to identify the number of unique structure clusters. This data abstraction is further described in Fig. S1. (*G*) We repeated steps (*E*) and (*F*) until the number of structure clusters converged. (*H*) The structure clusters are projected into 2D space using metric MDS. By minimizing the stress function for the Euclidean distance matrix, MDS optimizes the positioning of the structure clusters (40,41). To see this figure in color, go online.

the size of that element (35). Biologically, significant variation is observed in stem length but stack elements are generally more conserved (33–35). Thus, we expect that basing our projections on this distance metric will capture important structure/function features in the ensembles. Our representation counts the number of inner loops and stacks and then positions that count according to the location of the outermost stack in the nestedness feature vector (Fig. S1). Stems and stacks with fewer than three basepairs can be optionally ignored to simplify particularly complex ensemble visualizations. We determine the nestedness representation for every structure in the map of conformational space and collapse the structures into clusters based on unique nestedness representations (Fig. 2 *F*). Metric multidimensional scaling (MDS) projects the structure clusters into 2D space by finding the positioning of points in 2D space that best recapitulates the original Euclidean distances calculated from the structure cluster representations (Fig. 2 *H*) (40,41). MDS calculates the Euclidean distance matrix for n-dimensional data, d_{ij}. Initial positions for the data points, x, are set in 2D space, i and j. The initial positions are determined from projection onto the first two eigenvectors from eigen decomposition on the distance matrix. Based on this configuration, MDS evaluates the stress function in Eq. 2 (40,41). The data points are reconfigured in the direction of steepest descent. This process is repeated to minimize the stress function (40,41). Minimization of the stress function finds the configuration with the smallest residual sum of squares when compared with the original distance matrix (40,41). As a result, MDS yields a 2D embedding of the data points (used for visualization) that optimally reflects the pairwise distances between data points as computed within the original n-dimensional data, as follows:

$$\text{Stress} = \sqrt{\sum_{ij} \frac{\left(d_{ij} - \|x_i - x_j\|\right)^2}{\sum d_{ij}^2}}. \qquad (2)$$

Projection of the wild-type RNA

For the wild-type RNA, we recommend generating 1000 structures using Boltzmann-weighted stochastic sampling (42–45) (Fig. S2 *A*). SHAPE data can be included to direct the ensemble prediction (28,46). Each structure from the wild-type ensemble was converted into our nestedness representation. We then compute the frequency of structures that belong to each structure cluster (Fig. S2 *B*), and these frequencies are then scaled as relative diameters for each bubble in the resulting plot (Fig. S2 *C*). Bubbles are colored according to their 2D distances, where groups of similar clusters are closer on the viridis color scale in matplotlib (47). If any sampled structure in this plot does not match existing clusters in the map, the structure is added to the closest cluster based on Euclidean distance. As described here, the wild-type RNA is projected onto the map of conformational space. For comparison between mutants or between the same RNA in different environments, the same map of conformational space is used (as opposed to recomputing a new map for every comparison). This results in a stable space for projecting new ensembles of interest. In the interactive visualization output for EnsembleRNA, we include a measure of diversity for each structure cluster to allow the user to get a sense of the similarity of the structures clustered. This measure compares the frequency of the most common structure (maximum cluster frequency) and the average Jaccard similarity (48) between the binary representations of structures (minimum cluster correlation). Thus, if every structure in a cluster is unique, the value is 1; otherwise diversity is the fraction of nonunique structures.

EnsembleRNA package and webserver

A Python package (https://www.python.org/), EnsembleRNA, was created for the visualization of RNA structural ensembles. The package produces bubble charts for the map of conformational space and the wild-type RNA, and allows for comparison between structural ensembles. The package is available at http://ribosnitch.bio.unc.edu/software. Supporting Materials and Methods contains additional information on usage, troubleshooting EnsembleRNA, and tutorials.

In vitro SHAPE treatment

SHAPE-MaP experiments were performed in vitro (37). We obtained a clone of *β-actin* mRNA (SC319328; OriGene, Rockville, MD) and directly PCR-amplified the coding sequence with a 5′ primer containing the T7 promoter for in vitro transcription (Q5 Site-Directed Mutagenesis Kit and T7 RNA Polymerase from New England BioLabs, Ipswich, MA). To remove DNA after transcription, we treated the reaction with TURBO DNase for 15 min at 37°C (ThermoFisher Scientific, Waltham, MA). Standard bead cleanup was performed between each step (Ampure XP; Beckman Coulter, Brea, CA). The transcribed RNA was folded at 37°C in buffer containing 100 mM Na-HEPES, pH 8.0, 100 mM NaCl, and 10 mM $MgCl_2$. One μg of RNA was treated for 5 min with either 10% dimethyl sulfoxide (DMSO) or DMSO containing the RNA modifying agent 1-methyl-7-nitro-isatoic anhydride (1M7) at a final concentration of 10 mM.

In vivo SHAPE treatment

We performed in vivo SHAPE-MaP experiments for *β-actin* in the 1000 Genome Cell Lines GM07037 and GM12003 (37), obtained from the NIGMS Human Genetic Cell Repository at the Coriell Institute for Medical Research (https://www.coriell.org/). Approximately 50,000,000 cells were collected by centrifugation, resuspended in 1 mL of folding buffer (as in in vitro SHAPE protocol) supplemented with 400 U murine RNase inhibitor, and sonicated three times at 10% power for 10 s (Sonic Dismembrator Model 500; Thermo Fisher Scientific). These samples were incubated at 37°C for 10 min, after which either DMSO (10% final concentration) or 500 mM 1M7 in DMSO (final concentration 30 mM) was added for 5 min with three separate additions. RNA was isolated with Trizol reagent (ThermoFisher Scientific), followed by treatment with TURBO DNase and removal of the majority of ribosomal RNA (RiboMinus Eukaryote System v2; Life Technologies, Carlsbad, CA).

SHAPE data collection and analysis

For all samples, we performed reverse transcription with the specialized reverse transcription conditions for SHAPE-MaP and random nonamer primers (37). The transcription reactions were purified via Ampure XP beads (Beckman Coulter) or G50 columns (GE Healthcare Life Sciences, Little Chalfont, Buckinghamshire, UK), and dsDNA was made by second strand synthesis (NEBNext mRNA Second Strand Synthesis Module; New England BioLabs). To prepare libraries, we used the Nextera or Nextera XT kits (Nextera DNA Sample Preparation Kit, Nextera XT DNA Sample Preparation Kit and Index Kits; Illumina, San Diego, CA). Sequencing for the in vitro samples was performed on the HiSeq 2500 (Illumina) as paired-end, 50-read multiplex runs. Sequencing for the in vivo samples was performed on the HiSeq 2500 as paired-end, 100-read multiplex runs. Analysis was performed with the ShapeMapper pipeline (37) using either *β-actin* mRNA (NM_001101) to align sequences derived from the in vitro samples or the entire genome (hg38) to align sequences derived from the in vivo alignment. The *β-actin* data are in the file SNRNASM (see Data S1). SHAPE traces for the wild-type *V. vulnificus add* riboswitch mutate-and-map experiments were obtained from the publicly available RNA Mapping Database (22,49,50). To normalize the SHAPE-MaP data, scaled background reactivities were subtracted from the plus reagent reaction reactivities. A multiplier was used to fit the resulting distribution of values to the distribution of values for the normalized reactivities of a reference mRNA.

β-actin RNA structural modeling

An RNA/protein complex was generated from a starting model of two DNA strands bound to the KH34 protein (37). A custom Python script was used to convert the DNA strands to the appropriate RNA nucleotide sequence. The resulting RNA/protein complex was equilibrated by discrete molecular dynamics (DMD) simulations (51–53) to accommodate the zipcode binding regions of the RNA strands. The remaining regions of the RNA strands were modeled using coarse-grained DMD simulations (42) in which each nucleotide was represented as three pseudo-atoms corresponding to the phosphate backbone, sugar group, and nucleobase. With the replica exchange approach, we efficiently sampled RNA conformations by utilizing replicas of the same RNA system in parallel at different temperatures. Replicas were allowed to exchange simulation temperatures periodically based on a Monte Carlo algorithm. The replica exchange DMD simulations were run for 50 ns with replica temperatures of 0.200, 0.225, 0.250, 0.270, 0.300, 0.333, 0.367, and 0.400 with units kcal/(mol*kB). Free energy bonuses were incorporated between nucleotides to model the in vivo basepairing interactions. To select the final RNA model, we used a hierarchical clustering analysis based on the pairwise root mean square deviation of the phosphates and the potential energy as determined by the DMD force field. The coarse-grained RNA model was reconstructed to an all-atom model to combine with the KH34 protein system. We then equilibrated the entire RNA/protein complex using all-atom DMD simulations at a temperature of 0.4 kcal/(mol*kB) and included static constraints on the protein and harmonic constraints on the zipcode binding regions of the RNA strand.

In vitro model

We incorporated the in vitro secondary structure as constraints in coarse-grained replica exchange DMD simulations, using the same settings as those in the in vivo RNA system. We then performed an root mean square deviation-based clustering analysis to determine the centroid and reconstructed an all-atom model at a temperature of 0.4 kcal/(mol*kB).

RNA dynamics

The dynamics of the 2′ hydroxyl groups of the in vitro and in vivo RNA strands were calculated using the root mean square fluctuation (RMSF) with the Wordom software package (http://wordom.sourceforge.net/) (43). RMSF calculations were performed on three 100-ns DMD simulations at a temperature of 0.4 kcal/(mol*kB) for both RNA systems. The in vivo system included static constraints on the protein and harmonic constraints on the zipcode binding protein-interacting regions of the RNA. We calculated the mean using 3-nucleotide windows and SD of the RMSF based on the three DMD simulations for each system.

RESULTS

Generating a robust 2D representation of an RNA structural ensemble

Our first goal in creating a visualization of a structural ensemble was to establish a robust and consistent 2D representation of the conformational space of RNA. Traditionally, principal component analysis is used to identify two Eigenvectors for projection (24,25). One challenge with this approach is that the first three Eigenvectors often fail to capture enough variance to detect major structural elements (44). If a conformation change is predicted, this limitation of principal component analysis makes it difficult to understand the relative differences in the ensemble. A second challenge is determining which structural features to highlight in the representation to capture important biological aspects of the ensemble. Selecting features to highlight requires picking a specific structural distance representation, which can affect the interpretation as much as which Eigenvectors are used for projection. We propose an approach that provides a stable and robust visualization while also capturing important biological features (e.g., the on- and off-conformation of the *add* riboswitch in Fig. 1 *B*).

Our approach is summarized in Figs. 2 and S2. We begin by computing the partition function of the wild-type RNA sequence and all single point mutants. From these partition functions, we select the RNAs that are maximally different, as determined by Shannon entropy and hierarchical clustering on basepairing probability (36,39). From these partition functions, we sample the Boltzmann suboptimal ensemble and use these structures as the basis to build our visualization (25). This strategy effectively allows us to more comprehensively sample the suboptimal ensemble and the strategy does not depend on the approach used to compute the partition function. The visualization creates a stable space for the comparison of structural ensembles using mutations to explore the possible conformations that an RNA may take (Fig. 1 *B*). Data abstraction identifies clusters of similar structures that likely have similar function. This cluster representation reduces the map size, thereby creating a more accurate and interpretable visualization of secondary structure. Projecting the structure clusters into two dimensions using metric MDS optimizes their distances (40,41). This approach enables easy interpretation of the visualization, in which clusters that are farther apart are more different. We can project the RNA ensemble of interest onto this space by varying the size of cluster bubbles based on the number of structures that belong to that cluster (Fig. 1 *B*). Experimental structure probing data can be included to guide the ensemble prediction (45). This method is further described in the Materials and Methods.

Detecting RNA structure change induced by ligand binding

The *add* riboswitch is found in the 5′UTR of an mRNA that codes for adenosine deaminase (20,54). This riboswitch forms two distinct conformations that control translation of the adjacent coding region (20,54). The adenine-unbound conformation represses translation, and the adenine-bound conformation activates translation. Fig. 1 *A* shows the accepted secondary structures for the unbound and bound states as determined by crystallography and NMR (54). These secondary structures represent only two of several possible conformations that the riboswitch may adopt in the cell (28,46). Indeed, the map of conformational space produced by our visualization explores a total of five

possible structure clusters including the two accepted conformations (Fig. 1 *B*). This visualization produces a separation in 2D space between conformations that can bind adenine and activate translation and conformations that cannot bind adenine.

The structural difference induced by ligand binding for the *add* riboswitch is particularly well suited for the application of SHAPE data. Without experimental data to guide structure prediction algorithms, the accepted bound conformation dominates (Fig. 3 *A*), and differences in structure that result from changes in environment cannot be discerned. However, including SHAPE data in the ensemble prediction algorithms reveals differences in the *add* riboswitch structure with and without ligand (Fig. 3, *B* and *C*). In each ensemble, the respective structure observed in crystallography and NMR dominates. Thus, our visualization approach combined with SHAPE-directed structural modeling captures key structural features of the ensemble (20,54).

Observing regional structure differences in vitro and in vivo

β-actin is a cytoskeletal protein involved in cell motility and structure (15). The advent of high throughput structure probing methods such as SHAPE-MaP has only recently allowed us to collect information on larger RNAs such as the ~2-kb *β-actin* mRNA (37). Structure probing data are collected for RNA in the presence of cellular components, e.g., RNA-binding proteins (in vivo), and for free RNA (in vitro) (55). Thus, it is possible to detect structural differences in long mRNAs caused by differences in environments, such as the presence of ribosomes or RNA-binding proteins in the cell (56,57). Therefore, we performed SHAPE-MaP structure probing experiments on the *β-actin* mRNA present in in vitro and in vivo environments (Fig. 4).

Because we are specifically interested in differences between the two environments (in vivo and in vitro), we compute the windowed SHAPE correlation coefficient between the two data sets and plot this correlation in Fig. 4 *A* for a range of window sizes (40–140 nucleotides). Overall, we observe high correlation between the two data sets for a majority of the mRNA's span, with a mean correlation coefficient of 0.88. This result can be seen clearly in Fig. S3, in which we plot raw data for a highly similar window in the coding region of the gene. We begin our structural analysis by performing SHAPE-directed Boltzmann stochastic sampling of nucleotides 200–400, which we identified as having high in vitro to in vivo correlation (Fig. S3). We expect to observe only small changes in the stochastic sampling because the SHAPE data in this region are highly similar. As expected, the visualization confirmed only small differences, but it identified a remarkably complex ensemble with 24 structural clusters (Fig. S4). This result agrees with the high median SHAPE data (Fig. 4, *B* and *C*) observed for this region; high median SHAPE is correlated with higher ensemble entropy, i.e., multiple alternative conformations (37).

The region with the lowest correlation is at the 3′ end of the mRNA. The in vitro-probed mRNA was transcribed in the absence of a polyA polymerase, therefore it was not polyadenylated, which likely explains the differences near the 3′ end because the in vivo mRNA is most likely polyadenylated (and 5′-capped). The region of difference we chose to further characterize structurally occurs 3′ of the stop codon. This region in the mRNA contains functional elements known as the Zipcode Protein Binding Protein Sites (ZPBS1 and ZPBS2). Binding of the zipcode binding protein (ZBP1) mediates mRNA localization and translation, hence the name of the protein (58,59). We used our ensemble visualization approach to characterize the in vivo

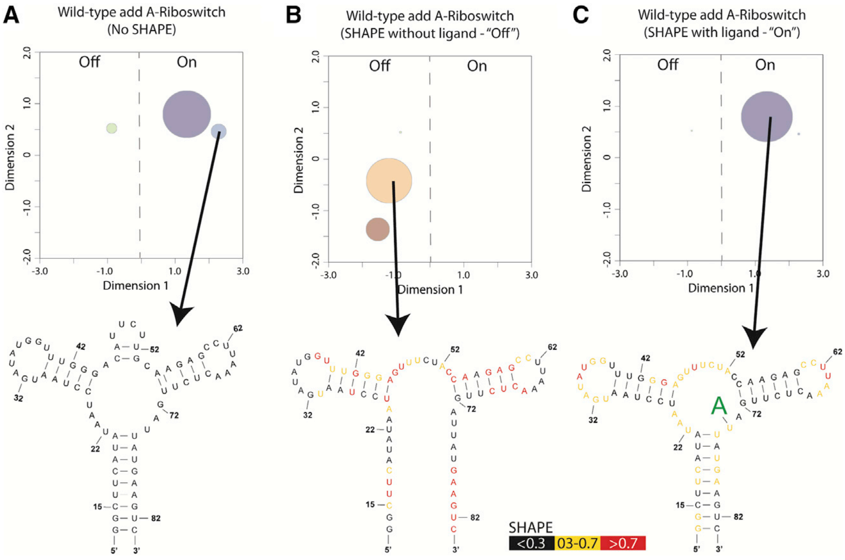

FIGURE 3 Visualization of bound and unbound states of the *V. vulnificus add* adenine riboswitch. (*A*) Projection of the predicted wild-type ensemble without SHAPE data favors the experimentally determined on-conformation (*left*). However, alternative conformations are still present (*right*). (*B*) When the ensemble generation is guided by SHAPE experiments conducted without ligand, off-conformations are favored in the projection (*left*). Particularly, the experimentally confirmed off-structure is the most populated conformation. (*C*) When SHAPE data are collected in the presence of ligand, the experimentally confirmed on-conformation (*right*) is preferred in the projection (*left*). Both SHAPE data sets (with and without ligand) are publicly available in the RNA Mapping Database (32,48,91). To see this figure in color, go online.

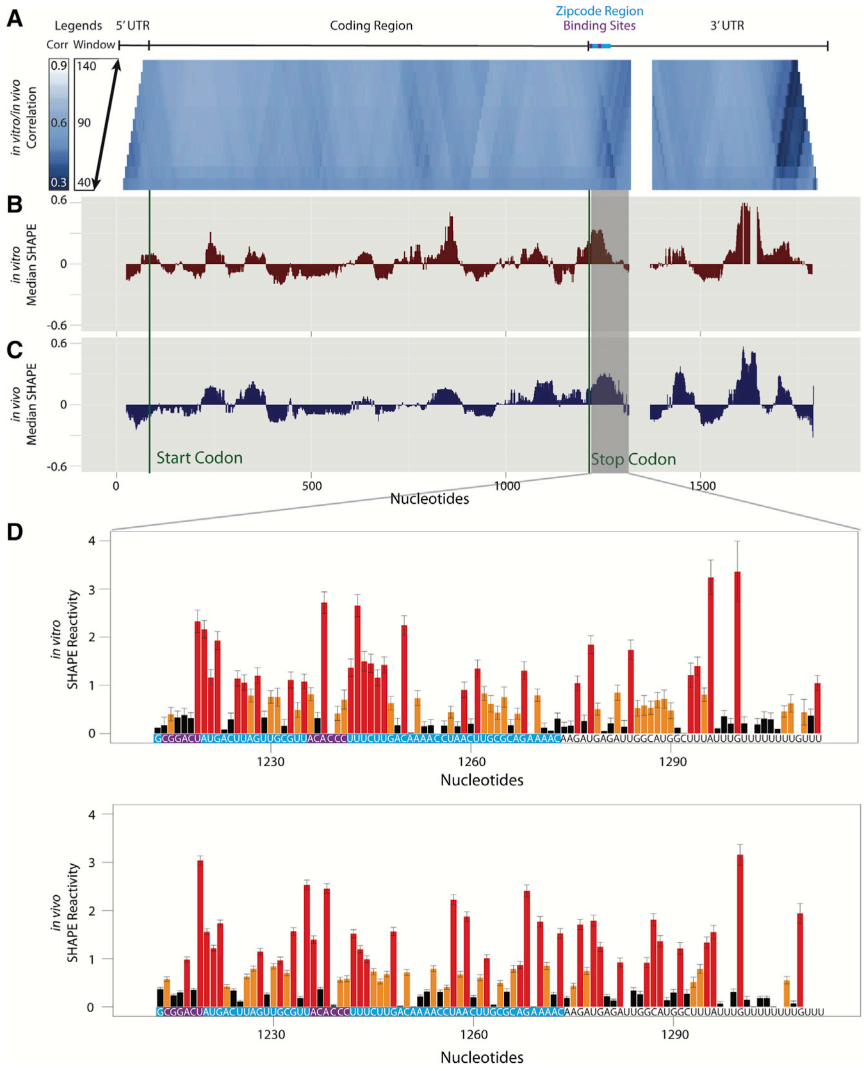

FIGURE 4 Comparison of in vitro and in vivo structure for the human *β-actin* mRNA. (*A*) We calculated the Pearson correlation in windows between the SHAPE reactivities collected in vitro and in vivo for the *β-actin* mRNA. For each step of the trapezoid from bottom to top, the window size increases by five nucleotides from 40 to 140. High correlation (*white*) corresponds to areas that are similar in structure and low correlation (*blue*) corresponds to areas that are different in structure. The distances from the median SHAPE value for (*B*) in vitro and (*C*) in vivo *β-actin* were calculated in 50-nucleotide windows. Segments with reactivities above the median are less structured than segments with reactivities below the median. The gray panel highlights a region in which the SHAPE reactivity differs between in vitro and in vivo. (*D*) This difference is seen in the SHAPE traces for in vitro (*top*) and in vivo trace (*bottom*). Structure probing was performed using the high throughput SHAPE-MaP technique. Red nucleotides correspond to high SHAPE reactivity, yellow corresponds to medium reactivity, and black corresponds to low reactivity. The ZBP1-binding region (*bright blue*) and two zipcode binding protein-interacting sites (*purple*) are labeled above the windowed correlation and at the bottom of the SHAPE traces. The overlay for the SHAPE traces is in Fig. S5. To see this figure in color, go online.

conformational rearrangements occurring in ZPBS1 and ZBPS2 within the ZBP1 binding region of the mRNA and to understand these rearrangements in the context of this region's function. The 54-nucleotide region we model below was previously identified as necessary and sufficient for localization of *β-actin* mRNA to the cell periphery (58,59). We therefore decided to specifically focus on the ensemble structure of this region.

Boltzmann stochastic sampling for the ZBP1 binding regions in vivo visualized using our approach revealed a shift in the structural ensemble away from the preferred in vitro conformation toward an alternative conformation (Fig. 5, *A* and *B*). Nonetheless, the dominant conformation in vitro (Fig. 5 *A*) is still significantly populated in vivo (Fig. 5 *B*). Thus, our visualization suggests a more complex ensemble of conformations in vivo. To further understand the structural context of the shift in ensemble, we visualized the secondary structure medoid for each of the largest structure clusters in vivo and in vitro. Although in both conformations the Zipcode Binding Protein Sites (ZBPS) are unpaired, in vivo the dominant confirmation shows ZBPS1 and ZBPS2 in a contiguous unpaired region, consistent with the larger in vivo SHAPE values. Importantly, the SHAPE reagent is not a footprinting reagent and is only minimally affected by nucleotide accessibility (60,61). Thus, it is not surprising that we observed higher SHAPE values surrounding the ZBPS. In fact, the ZBP1 is divalent, and it has been shown to simultaneously bind the two ZBPS motifs separated by a linker portion of the RNA, although the precise occupancy of the second site is not known (37,62). Nonetheless, binding to this region is essential for correct *β-actin* mRNA localization and translational control (63,64). To accommodate the ZBP1 protein, the RNA likely has to become more open and flexible, consistent with the higher SHAPE data we observed.

To further understand the in vivo structural rearrangement, we performed molecular simulations of apo and bound mRNA conformations (Fig. 5, *C* and *D*). By using the secondary structure as initial constraints, we aimed to estimate the root mean square fluctuations (RMSFs) of the

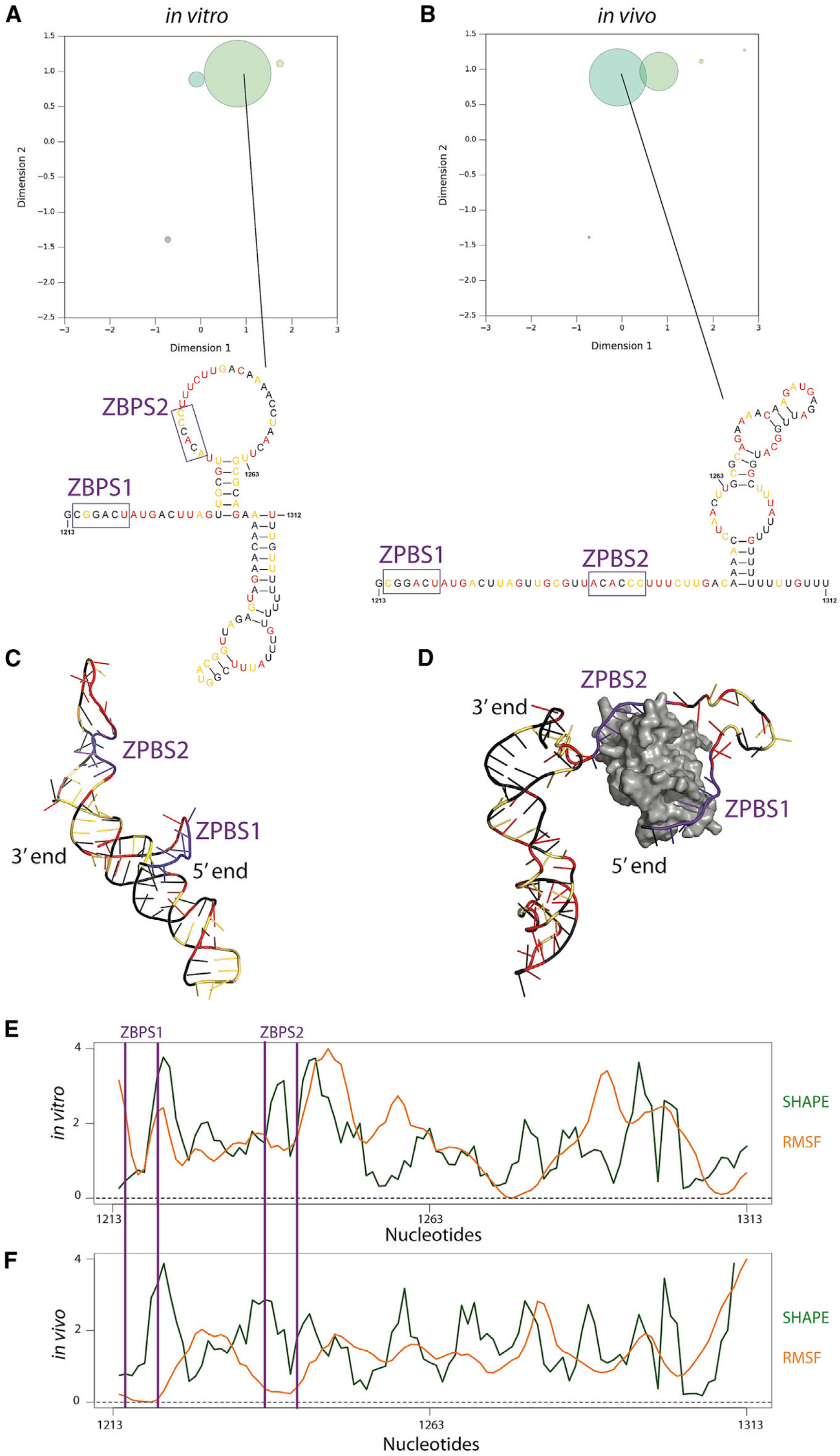

FIGURE 5 Ensemble visualization for in vitro and in vivo human *β-actin* mRNA. Generation of structures for the *β-actin* mRNA ensemble was guided by the in vitro and in vivo SHAPE data. We compared the (*A*) in vitro and (*B*) in vivo ensembles for the region where SHAPE reactivities were expected to be different. The ensemble visualization reveals a large shift away from the dominant structure in vitro toward a second structure in vivo. We visualized the second structure for the medoid in each of the largest structure clusters. These nucleotides form different structures in vitro and in vivo. The region that differs includes the zipcode region with the two ZBP1 binding sites (*purple*). (*C*) The 3D structure for *β-actin* in vitro was modeled using molecular dynamics simulations without ZBP1. (*D*) The 3D structure for *β-actin* in vivo was modeled with the ZBP1 (in *gray*). For both 3D models, the ZBP1 binding regions are highlighted in purple. Red nucleotides correspond to high SHAPE reactivity, yellow correspond to medium reactivity, and black corresponds to low reactivity in (*A*–*D*) and (*E*). Comparison of SHAPE reactivity (*green*) and normalized RMSF (*orange*) for *β-actin* in vitro largely follow the same pattern. (*F*) Comparison of SHAPE reactivity and RMSF for *β-actin* in vivo also largely follow the same pattern. The SHAPE reactivities and RMSF values are averaged across a 3-nucleotide moving window. The RMSF is calculated from the 3D structural models. ZPB1 binding sites for (*E*) and (*F*) are boxed in purple. Fig. S6 includes further comparisons between in vitro and in vivo SHAPE reactivity and RMSF. To see this figure in color, go online.

RNA backbone. We show these data for the apo and bound simulations in Fig. 5, *E* and *F*, overlaid with the in vitro and in vivo SHAPE data, respectively. We observed qualitative agreements between the experimental SHAPE data and the simulations, suggesting these molecular models captured overall aspects of the conformational ensemble.

One important aspect of these comparisons, especially in the case of the in vivo data, is that the SHAPE data are an ensemble average over all of the *β-actin* mRNA molecules in the cell. Because ZBP1 binding represses translation, some message molecules are likely not bound by ZBP1, a situation that may explain why a shift to multiple

conformations is observed in vivo as opposed to observing only the bound conformation. Nonetheless, these data demonstrate the value of visualizing the structural ensemble to explain structure/function relationships in an mRNA.

DISCUSSION

RNA structure is the key component of cellular function in highly specific instances; the ribosome's unique catalytic core is a prime example of the role of a specific RNA structure in performing protein synthesis (65–69). Generally, however, the functions of structures in messenger RNAs are poorly understood, except for a few cases, such as the iron responsive element (22,70) and the histone stem loop (71–73), in which single structures are essential for function. Other than ribosomal RNA, no RNA larger than 1 kb, including mRNAs, is known to fold into a unique, well-defined conformation (65,67–69,74,75). Still, although large RNAs do not adopt single conformations, specific regions do fold into complex 3D structures. One example is riboswitches in bacteria (Fig. 1). Although riboswitches are considered to be structured (i.e., they can be crystallized), riboswitches adopt multiple conformations that lead to different functions (7). Because RNAs such as riboswitches have evolved to form multiple conformations to function, it is essential to consider the suboptimal ensemble when considering structure in messenger RNAs (7).

Our approach to visualizing the suboptimal ensemble is designed to resolve some of the longstanding problems with obtaining a stable projection that allows comparisons of ensembles. A priori, this visualization approach requires sampling the entire suboptimal space to identify good principal components. For any biologically relevant RNA, such sampling rapidly becomes computationally intractable because the number of suboptimal conformations increases exponentially with length (76,77). Thus, our approach is empirical (Fig. 2) and relies on rapid sampling of suboptimal ensembles for single point mutants of the RNA (78). Combined with multidimensional scaling and a shape-based abstraction (25,76), our maps have the desired properties of stability and they enable comparison of different ensembles. The stability of our visualization and the complexity of unstructured regions are best illustrated in Figs. S3 and S4.

The main biological motivation for our approach is the need to visualize changes in the ensemble caused by environment. Our results on the *V. vulnificus add* riboswitch leverage the empirical relationship between SHAPE reactivity and the free energy of folding to recapitulate the apo and bound RNA ensembles (Fig. 3). Importantly, the goal of these visualizations is to facilitate the understanding of a complex process by approximating the specific abundance of each conformation in an ensemble. Moreover, we aim to extract biological insight from the ensemble calculation; for the *V. vulnificus add* riboswitch, our visualization of the ensemble model recapitulates the understanding of this system in an easily interpreted diagram. The riboswitch, a smaller fragment of a larger bacterial mRNA, is a relatively straightforward example. This is not the case for complex full-length eukaryotic mRNAs that tend to be much more highly regulated and structurally sensitive to their environments (38,79). Whether prokaryotic or eukaryotic, it is clear that mRNAs are in integral part of cellular regulation (38).

Our analyses of a full-length human mRNA in vivo and in vitro revealed some of the complexities associated with interpreting structures in large RNAs. We observed, in both conditions, regions of high (unstructured) and low (structured) median SHAPE (56), results consistent with locally structured regions. Overall, the high similarity between in vivo and in vitro SHAPE data suggests that the mRNA is not globally affected by its environment, but, instead, specific regions are affected by endogenous molecule binding. Local structure is the case for the ZPB1 binding region in the 3′ UTR of *β-actin*, which we visualized using our ensemble approach (Fig. 5).

A significant result of this analysis is the median windowed SHAPE, which overall appeared higher in vivo relative to in vitro for the ZPB1-binding region. This result may seem counterintuitive, as the ZBP1 would be expected to protect the RNA from the 1M7 reagent. Although protein binding is detectable by SHAPE comparisons in vitro to in vivo (56,80), SHAPE chemistry is not a traditional footprinting technique (44,81–83). Thus, it is likely that the majority of differences in the SHAPE reactivity in this region are due to a conformational rearrangement due to protein binding, and not the footprint of the protein.

Our model (Fig. 5, *A* and *B*) successfully reports a shift in the ensemble, but the model does not suggest a totally dominant alternative in vivo conformation. This restriction is in contrast to the *add* riboswitch, in which ligand excess shifts the ensemble to almost completely the on-conformation (Fig. 3 *C*). It is important not to overinterpret the relative ratios of the two dominant conformations proposed for the ZBP1-binding region modeled in Fig. 5 *B*. However, the model is consistent with our expectation of a mixed population of ZBP1-bound and unbound *β-actin* mRNA. Also, the fact that the ZBP1 has two binding sites and these sites are not always simultaneously occupied (37,84) is an additional aspect that our model cannot currently describe. Thus, our visualization accurately represents the likely state of the population of *β-actin* mRNAs in the cell, but still requires biological knowledge to be fully interpretable.

We performed constrained molecular dynamics simulations of the two proposed structural models of *β-actin* mRNA to determine if the models agreed qualitatively with the SHAPE data. Because SHAPE chemistry measures backbone flexibility (81,85), we report RMSFs for both models in Fig. 5, *E* and *F*. For the ZBP1-binding region between ZBPS1 and ZBPS1, the agreement between the simulation and SHAPE data is better for the in vitro model compared to the in vivo simulation. For the in vivo model,

we constrained both ZBPS1 and ZBPS2 to the binding pockets, which explains the low flexibility of ZBPS1 and ZBPS2. The higher SHAPE data for these two binding sites in vivo are consistent with a significant subset of mRNAs being unbound, which agrees with our ensemble model that suggested a further opening of the structure.

In summary, we have developed a computationally based visualization approach that faithfully represents ensemble mRNA populations and the effects of environment on the ensembles. The *β-actin* mRNA and the *V. vulnificus add* riboswitch are two well-characterized systems in which ensemble visualization improves the interpretation of environmentally imposed structural differences. By releasing a software package to create these visualizations easily, we encourage the RNA folding community to simulate more than just minimum free energy structures and to explore the suboptimal ensemble for all mRNAs existing in a cell. It is not clear whether suboptimal alternative conformations are a necessary component of RNA function in the cell or a by-product of the rules that govern RNA folding (28,86–89). Regardless, structure ensembles are a thermodynamic reality of RNAs and are accommodated as a feature of their function.

SUPPORTING MATERIAL

Supporting Materials and Methods, EnsembleRNA software documentation, nine figures, and one data file are available at http://www.biophysj.org/biophysj/supplemental/S0006-3495(17)30570-2.

AUTHOR CONTRIBUTIONS

A.L., C.T.W., L.L., D.G., B.W., and N.V.D. designed the experiments. C.T.W. designed the visualization algorithm. L.L. performed SHAPE-MaP experiments. B.W. performed molecular dynamic simulations. A.L., C.T.W., L.L., and B.W. analyzed data. A.L, C.T.W., L.L., and B.W. wrote the manuscript.

ACKNOWLEDGMENTS

We thank Jeffrey A. Chao for supplying the initial ZBP1 structural model for our MD simulations.

This work was supported by the National Institutes of Health (NIH) under grant Nos. HL111527, GM101237, and HG008133 to A.L., grant Nos. R01 GM123238-01, 1R01GM123247, and R01 GM064803-12 to N.V.D., and grant No. 3R01GM080742-08S1 to B.W. L.L. was supported by an American Cancer Society – Lee National Denim Day Postdoctoral Fellowship, grant No. PF-15-133-01-RMC.

REFERENCES

1. Chauhan, S., and S. A. Woodson. 2008. Tertiary interactions determine the accuracy of RNA folding. *J. Am. Chem. Soc.* 130:1296–1303.
2. Shcherbakova, I., S. Mitra, …, M. Brenowitz. 2008. Energy barriers, pathways, and dynamics during folding of large, multidomain RNAs. *Curr. Opin. Chem. Biol.* 12:655–666.
3. Mitra, S., A. Laederach, …, M. Brenowitz. 2011. RNA molecules with conserved catalytic cores but variable peripheries fold along unique energetically optimized pathways. *RNA.* 17:1589–1603.
4. Sinan, S., X. Yuan, and R. Russell. 2011. The Azoarcus group I intron ribozyme misfolds and is accelerated for refolding by ATP-dependent RNA chaperone proteins. *J. Biol. Chem.* 286:37304–37312.
5. Noller, H. F. 2005. RNA structure: reading the ribosome. *Science.* 309:1508–1514.
6. Sclavi, B., E. Zaychikov, …, H. Heumann. 2005. Real-time characterization of intermediates in the pathway to open complex formation by *Escherichia coli* RNA polymerase at the T7A1 promoter. *Proc. Natl. Acad. Sci. USA.* 102:4706–4711.
7. Ritz, J., J. S. Martin, and A. Laederach. 2013. Evolutionary evidence for alternative structure in RNA sequence co-variation. *PLOS Comput. Biol.* 9:e1003152.
8. Kutchko, K. M., W. Sanders, …, A. Laederach. 2015. Multiple conformations are a conserved and regulatory feature of the RB1 5′ UTR. *RNA.* 21:1274–1285.
9. Thirumalai, D., and C. Hyeon. 2005. RNA and protein folding: common themes and variations. *Biochemistry.* 44:4957–4970.
10. Ponty, Y. 2008. Efficient sampling of RNA secondary structures from the Boltzmann ensemble of low-energy: the Boustrophedon method. *J. Math. Biol.* 56:107–127.
11. Eddy, S. R. 2009. A new generation of homology search tools based on probabilistic inference. *Genome Inform.* 23:205–211.
12. Das, R., L. W. Kwok, …, D. Herschlag. 2003. The fastest global events in RNA folding: electrostatic relaxation and tertiary collapse of the Tetrahymena ribozyme. *J. Mol. Biol.* 332:311–319.
13. Martin, J. S., M. Halvorsen, …, A. Laederach. 2012. Structural effects of linkage disequilibrium on the transcriptome. *RNA.* 18:77–87.
14. Shapiro, B. A., D. Bengali, …, J. C. Wu. 2001. RNA folding pathway functional intermediates: their prediction and analysis. *J. Mol. Biol.* 312:27–44.
15. Cordero, P., and R. Das. 2015. Rich RNA structure landscapes revealed by mutate-and-map analysis. *PLOS Comput. Biol.* 11:e1004473.
16. Delfosse, V., P. Bouchard, …, P. Legault. 2010. Riboswitch structure: an internal residue mimicking the purine ligand. *Nucleic Acids Res.* 38:2057–2068.
17. Lemay, J. F., and D. A. Lafontaine. 2007. Core requirements of the adenine riboswitch aptamer for ligand binding. *RNA.* 13:339–350.
18. Lemay, J. F., J. C. Penedo, …, D. A. Lafontaine. 2006. Folding of the adenine riboswitch. *Chem. Biol.* 13:857–868.
19. Lemay, J. F., G. Desnoyers, …, D. A. Lafontaine. 2011. Comparative study between transcriptionally- and translationally acting adenine riboswitches reveals key differences in riboswitch regulatory mechanisms. *PLoS Genet.* 7:e1001278.
20. Lemay, J. F., J. C. Penedo, …, D. A. Lafontaine. 2009. Molecular basis of RNA-mediated gene regulation on the adenine riboswitch by single-molecule approaches. *Methods Mol. Biol.* 540:65–76.
21. Bokinsky, G., and X. Zhuang. 2005. Single-molecule RNA folding. *Acc. Chem. Res.* 38:566–573.
22. Halvorsen, M., J. S. Martin, …, A. Laederach. 2010. Disease-associated mutations that alter the RNA structural ensemble. *PLoS Genet.* 6:e1001074.
23. Roh, J. H., L. Guo, …, S. A. Woodson. 2010. Multistage collapse of a bacterial ribozyme observed by time-resolved small-angle x-ray scattering. *J. Am. Chem. Soc.* 132:10148–10154.
24. Ding, Y., C. Y. Chan, and C. E. Lawrence. 2004. Sfold Web Server for statistical folding and rational design of nucleic acids. *Nucleic Acids Res.* 32:W135–W141.
25. Ding, Y., C. Y. Chan, and C. E. Lawrence. 2005. RNA secondary structure prediction by centroids in a Boltzmann weighted ensemble. *RNA.* 11:1157–1166.

26. Hamada, M., H. Kiryu, …, K. Asai. 2009. Prediction of RNA secondary structure using generalized centroid estimators. *Bioinformatics.* 25:465–473.
27. Waldispühl, J., and P. Clote. 2007. Computing the partition function and sampling for saturated secondary structures of RNA, with respect to the Turner energy model. *J. Comput. Biol.* 14:190–215.
28. Deigan, K. E., T. W. Li, …, K. M. Weeks. 2009. Accurate SHAPE-directed RNA structure determination. *Proc. Natl. Acad. Sci. USA.* 106:97–102.
29. Wilkinson, K. A., S. M. Vasa, …, K. M. Weeks. 2009. Influence of nucleotide identity on ribose 2′-hydroxyl reactivity in RNA. *RNA.* 15:1314–1321.
30. Cordero, P., J. B. Lucks, and R. Das. 2012. An RNA Mapping DataBase for curating RNA structure mapping experiments. *Bioinformatics.* 28:3006–3008.
31. Cheng, C. Y., F. C. Chou, …, R. Das. 2015. Consistent global structures of complex RNA states through multidimensional chemical mapping. *eLife.* 4:e07600.
32. Kladwang, W., P. Cordero, and R. Das. 2011. A mutate-and-map strategy accurately infers the base pairs of a 35-nucleotide model RNA. *RNA.* 17:522–534.
33. Shapiro, B. A. 1988. An algorithm for comparing multiple RNA secondary structures. *Comput. Appl. Biosci.* 4:387–393.
34. Rivas, E., and S. R. Eddy. 2001. Noncoding RNA gene detection using comparative sequence analysis. *BMC Bioinformatics.* 2:8.
35. Macke, T. J., D. J. Ecker, …, R. Sampath. 2001. RNAMotif, an RNA secondary structure definition and search algorithm. *Nucleic Acids Res.* 29:4724–4735.
36. Shannon, C. E. 1951. Prediction and entropy of printed English. *Bell Syst. Tech. J.* 30:50–64.
37. Siegfried, N. A., S. Busan, …, K. M. Weeks. 2014. RNA motif discovery by SHAPE and mutational profiling (SHAPE-MaP). *Nat. Methods.* 11:959–965.
38. Kutchko, K. M., and A. Laederach. 2016. Transcending the prediction paradigm: novel applications of SHAPE to RNA function and evolution. *Wiley Interdiscip. Rev. RNA.* 8:e1374.
39. Defays, D. 1977. An efficient algorithm for a complete link method. *Comput. J.* 20:364–366.
40. Abdi, H. 2007. Metric Multidimensional Scaling (MDS): Analyzing Distance Matrices. Sage, Thousand Oaks, CA.
41. Torgerson, W. 1952. Multidimensional scaling: I. Theory and method. *Psychometrika.* 17:401–419.
42. Dokholyan, N. V., S. V. Buldyrev, …, E. I. Shakhnovich. 1998. Discrete molecular dynamics studies of the folding of a protein-like model. *Fold. Des.* 3:577–587.
43. Ding, F., S. Sharma, …, N. V. Dokholyan. 2008. Ab initio RNA folding by discrete molecular dynamics: from structure prediction to folding mechanisms. *RNA.* 14:1164–1173.
44. Seeber, M., A. Felline, …, F. Fanelli. 2011. Wordom: a user-friendly program for the analysis of molecular structures, trajectories, and free energy surfaces. *J. Comput. Chem.* 32:1183–1194.
45. Quarrier, S., J. S. Martin, …, A. Laederach. 2010. Evaluation of the information content of RNA structure mapping data for secondary structure prediction. *RNA.* 16:1108–1117.
46. Ding, Y., C. Y. Chan, and C. E. Lawrence. 2006. Clustering of RNA secondary structures with application to messenger RNAs. *J. Mol. Biol.* 359:554–571.
47. Hunter, J. D. 2007. Matplotlib: A 2D graphics environment. *Comput. Sci. Eng.* 9:90–95.
48. Mathews, D. H. 2006. Revolutions in RNA secondary structure prediction. *J. Mol. Biol.* 359:526–532.
49. Mathews, D. H., and D. H. Turner. 2002. Experimentally derived nearest-neighbor parameters for the stability of RNA three- and four-way multibranch loops. *Biochemistry.* 41:869–880.
50. Mathews, D. H., M. D. Disney, …, D. H. Turner. 2004. Incorporating chemical modification constraints into a dynamic programming algorithm for prediction of RNA secondary structure. *Proc. Natl. Acad. Sci. USA.* 101:7287–7292.
51. Chao, J. A., Y. Patskovsky, …, R. H. Singer. 2010. ZBP1 recognition of β-actin zipcode induces RNA looping. *Genes Dev.* 24:148–158.
52. Shirvanyants, D., F. Ding, …, N. V. Dokholyan. 2012. DMD: an efficient and versatile simulation method for fine protein characterization. *J. Phys. Chem. B.* 116 (29):8375–8382.
53. Dokholyan, N. V., F. Ding, and E. A. Proctor. 2011. Discrete molecular dynamics. *WIREs Comput. Mol. Sci.* 1:80–92.
54. Serganov, A., Y.-R. Yuan, …, D. J. Patel. 2004. Structural basis for discriminative regulation of gene expression by adenine- and guanine-sensing mRNAs. *Chem. Biol.* 11:1729–1741.
55. Guo, C., S. Liu, …, F. T. Greenaway. 2013. ACTB in cancer. *Clin. Chim. Acta.* 417:39–44.
56. Spitale, R. C., P. Crisalli, …, H. Y. Chang. 2013. RNA SHAPE analysis in living cells. *Nat. Chem. Biol.* 9:18–20.
57. Smola, M. J., T. W. Christy, …, K. M. Weeks. 2016. SHAPE reveals transcript-wide interactions, complex structural domains, and protein interactions across the Xist lncRNA in living cells. *Proc. Natl. Acad. Sci. USA.* 113:10322–10327.
58. Smola, M. J., J. M. Calabrese, and K. M. Weeks. 2015. Detection of RNA-protein interactions in living cells with SHAPE. *Biochemistry.* 54:6867–6875.
59. Lawrence, J. B., and R. H. Singer. 1986. Intracellular localization of messenger RNAs for cytoskeletal proteins. *Cell.* 45:407–415.
60. Kislauskis, E. H., X. Zhu, and R. H. Singer. 1994. Sequences responsible for intracellular localization of β-actin messenger RNA also affect cell phenotype. *J. Cell Biol.* 127:441–451.
61. Wilkinson, K. A., E. J. Merino, and K. M. Weeks. 2006. Selective 2′-hydroxyl acylation analyzed by primer extension (SHAPE): quantitative RNA structure analysis at single nucleotide resolution. *Nat. Protoc.* 1:1610–1616.
62. Merino, E. J., K. A. Wilkinson, …, K. M. Weeks. 2005. RNA structure analysis at single nucleotide resolution by selective 2′-hydroxyl acylation and primer extension (SHAPE). *J. Am. Chem. Soc.* 127:4223–4231.
63. Patel, V. L., S. Mitra, …, J. A. Chao. 2012. Spatial arrangement of an RNA zipcode identifies mRNAs under post-transcriptional control. *Genes Dev.* 26:43–53.
64. Ross, A. F., Y. Oleynikov, …, R. H. Singer. 1997. Characterization of a β-actin mRNA zipcode-binding protein. *Mol. Cell. Biol.* 17:2158–2165.
65. Hüttelmaier, S., D. Zenklusen, …, R. H. Singer. 2005. Spatial regulation of β-actin translation by Src-dependent phosphorylation of ZBP1. *Nature.* 438:512–515.
66. Ban, N., P. Nissen, …, T. A. Steitz. 2000. The complete atomic structure of the large ribosomal subunit at 2.4 Å resolution. *Science.* 289:905–920.
67. Harms, J., F. Schluenzen, …, A. Yonath. 2001. High resolution structure of the large ribosomal subunit from a mesophilic eubacterium. *Cell.* 107:679–688.
68. Ramakrishnan, V. 2002. Ribosome structure and the mechanism of translation. *Cell.* 108:557–572.
69. Wimberly, B. T., D. E. Brodersen, …, V. Ramakrishnan. 2000. Structure of the 30S ribosomal subunit. *Nature.* 407:327–339.
70. Yonath, A. 2010. Hibernating bears, antibiotics and the evolving ribosome (Nobel Lecture). *Angew. Chem.* 49:4340–4354.
71. Ritz, J., J. S. Martin, and A. Laederach. 2012. Evaluating our ability to predict the structural disruption of RNA by SNPs. *BMC Genomics.* 13 (*Suppl 4*):S6.
72. Harris, M. E., R. Böhni, …, W. F. Marzluff. 1991. Regulation of histone mRNA in the unperturbed cell cycle: evidence suggesting control at two posttranscriptional steps. *Mol. Cell. Biol.* 11:2416–2424.

73. Pandey, N. B., and W. F. Marzluff. 1987. The stem-loop structure at the 3′ end of histone mRNA is necessary and sufficient for regulation of histone mRNA stability. *Mol. Cell. Biol.* 7:4557–4559.

74. Sun, J., D. R. Pilch, and W. F. Marzluff. 1992. The histone mRNA 3′ end is required for localization of histone mRNA to polyribosomes. *Nucleic Acids Res.* 20:6057–6066.

75. Berman, H. M., W. K. Olson, …, B. Schneider. 1992. The nucleic acid database. A comprehensive relational database of three-dimensional structures of nucleic acids. *Biophys. J.* 63:751–759.

76. Coimbatore Narayanan, B., J. Westbrook, …, H. M. Berman. 2014. The nucleic acid database: new features and capabilities. *Nucleic Acids Res.* 42:D114–D122.

77. Giegerich, R., B. Voss, and M. Rehmsmeier. 2004. Abstract shapes of RNA. *Nucleic Acids Res.* 32:4843–4851.

78. Sachs, A. B., P. Sarnow, and M. W. Hentze. 1997. Starting at the beginning, middle, and end: translation initiation in eukaryotes. *Cell.* 89:831–838.

79. Steffen, P., B. Voss, …, R. Giegerich. 2006. RNAshapes: an integrated RNA analysis package based on abstract shapes. *Bioinformatics.* 22:500–503.

80. Solem, A. C., M. Halvorsen, …, A. Laederach. 2015. The potential of the riboSNitch in personalized medicine. *Wiley Interdiscip. Rev. RNA.* 6:517–532.

81. McGinnis, J. L., Q. Liu, …, K. M. Weeks. 2015. In-cell SHAPE reveals that free 30S ribosome subunits are in the inactive state. *Proc. Natl. Acad. Sci. USA.* 112:2425–2430.

82. McGinnis, J. L., J. A. Dunkle, …, K. M. Weeks. 2012. The mechanisms of RNA SHAPE chemistry. *J. Am. Chem. Soc.* 134:6617–6624.

83. Brenowitz, M., M. R. Chance, …, K. Takamoto. 2002. Probing the structural dynamics of nucleic acids by quantitative time-resolved and equilibrium hydroxyl radical “footprinting”. *Curr. Opin. Struct. Biol.* 12:648–653.

84. Shcherbakova, I., S. Mitra, …, M. Brenowitz. 2006. Fast Fenton footprinting: a laboratory-based method for the time-resolved analysis of DNA, RNA and proteins. *Nucleic Acids Res.* 34:e48.

85. Kim, H. H., S. J. Lee, …, S. Yoo. 2015. Different motif requirements for the localization zipcode element of β-actin mRNA binding by HuD and ZBP1. *Nucleic Acids Res.* 43:7432–7446.

86. Herschlag, D., B. E. Allred, and S. Gowrishankar. 2015. From static to dynamic: the need for structural ensembles and a predictive model of RNA folding and function. *Curr. Opin. Struct. Biol.* 30:125–133.

87. Russell, R., X. Zhuang, …, D. Herschlag. 2002. Exploring the folding landscape of a structured RNA. *Proc. Natl. Acad. Sci. USA.* 99:155–160.

88. Solomatin, S. V., M. Greenfeld, …, D. Herschlag. 2010. Multiple native states reveal persistent ruggedness of an RNA folding landscape. *Nature.* 463:681–684.

89. Gracia, B., Y. Xue, …, R. Russell. 2016. RNA structural modules control the rate and pathway of RNA folding and assembly. *J. Mol. Biol.* 428:3972–3985.

90. Liu, Y., E. Holmstrom, …, Y. X. Wang. 2015. Synthesis and applications of RNAs with position-selective labelling and mosaic composition. *Nature.* 522:368–372.

91. Kladwang, W., C. C. VanLang, …, R. Das. 2011. A two-dimensional mutate-and-map strategy for non-coding RNA structure. *Nat. Chem.* 3:954–962.

92. Mathews, D. H., J. Sabina, …, D. H. Turner. 1999. Expanded sequence dependence of thermodynamic parameters improves prediction of RNA secondary structure. *J. Mol. Biol.* 288:911–940.

Biophysical Journal, Volume *113*

Supplemental Information

Comparative Visualization of the RNA Suboptimal Conformational Ensemble In Vivo

Chanin T. Woods, Lela Lackey, Benfeard Williams, Nikolay V. Dokholyan, David Gotz, and Alain Laederach

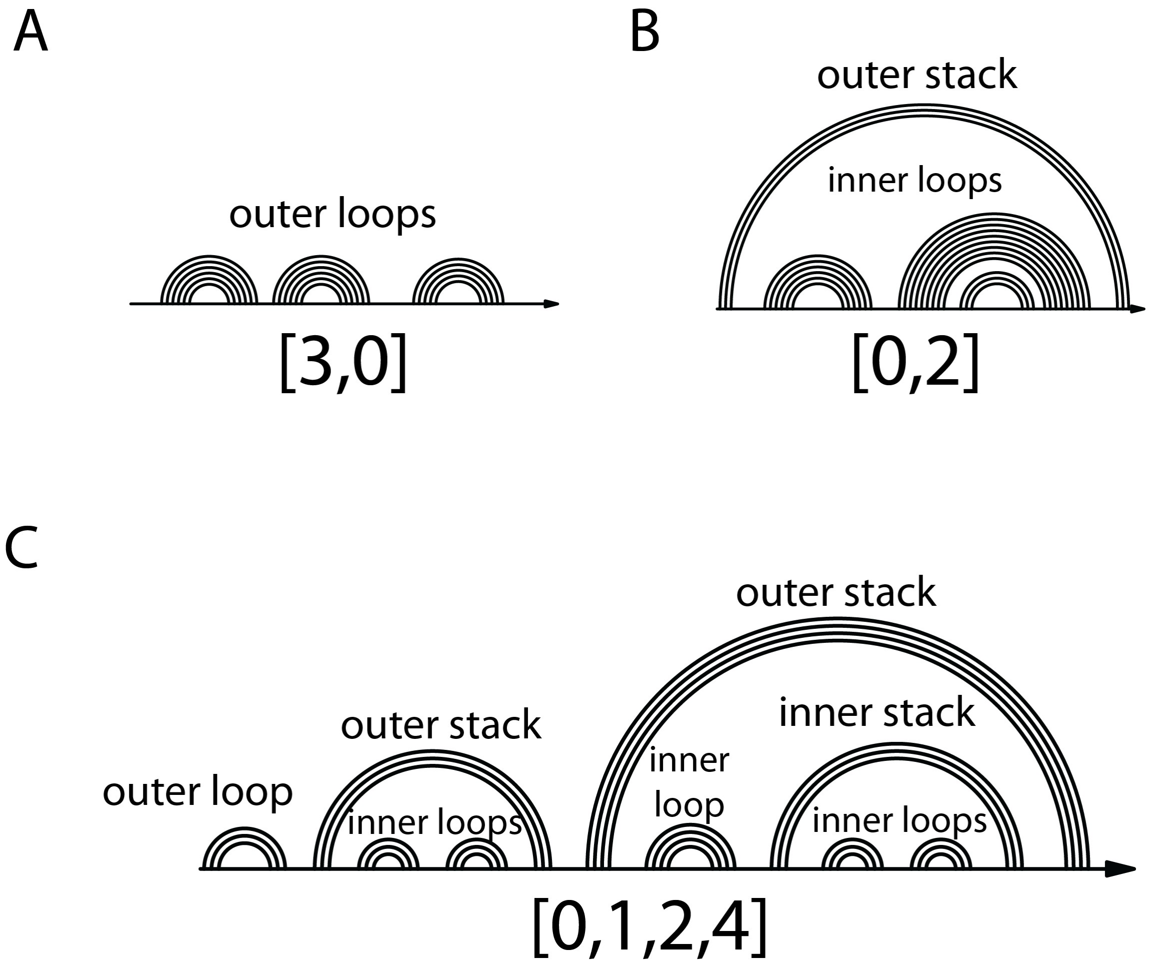

Figure S1. RNA structure abstraction and nestedness. We utilized RNAshapes abstraction to identify unique structure clusters (1). This abstraction assumes that the sizes of structural elements are less important than whether they are present. We used this method to create a numeric vector representation. This representation is based on the nestedness of RNA stacks and loops. A) If only outer loops are present in a structure, we place the number of outer loops in the first column. B) If an outer stack is present, we place the number of loops inside that stack in the n+1 column. C) For each outer loop or outer stack, we place the number of inside loops into the n+1 column.

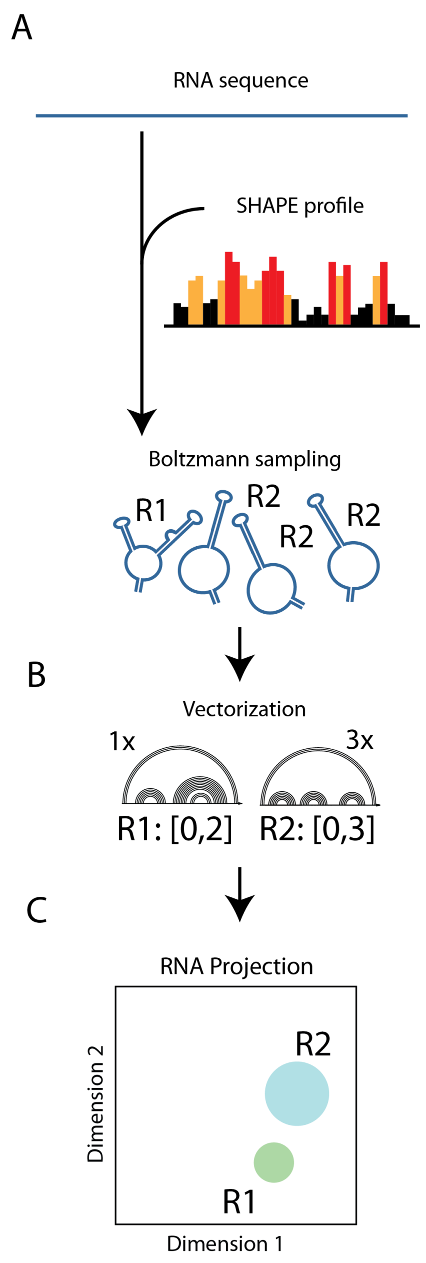

Figure S2. Projection of the reference RNA. A) We generated 1000 structures using Boltzmann-weighted suboptimal sampling from the reference RNA sequence (2). We used experimental structure data collected from selective 2'-hydroxyl acylation analyzed by primer extension (SHAPE) to guide the ensemble prediction (3, 4). Each structure was converted into our nestedness representation (Figure 2E). We retained the orientation of the points from the map of conformational space. The size of each bubble was varied based on the frequency for that structure cluster in the wild type ensemble.

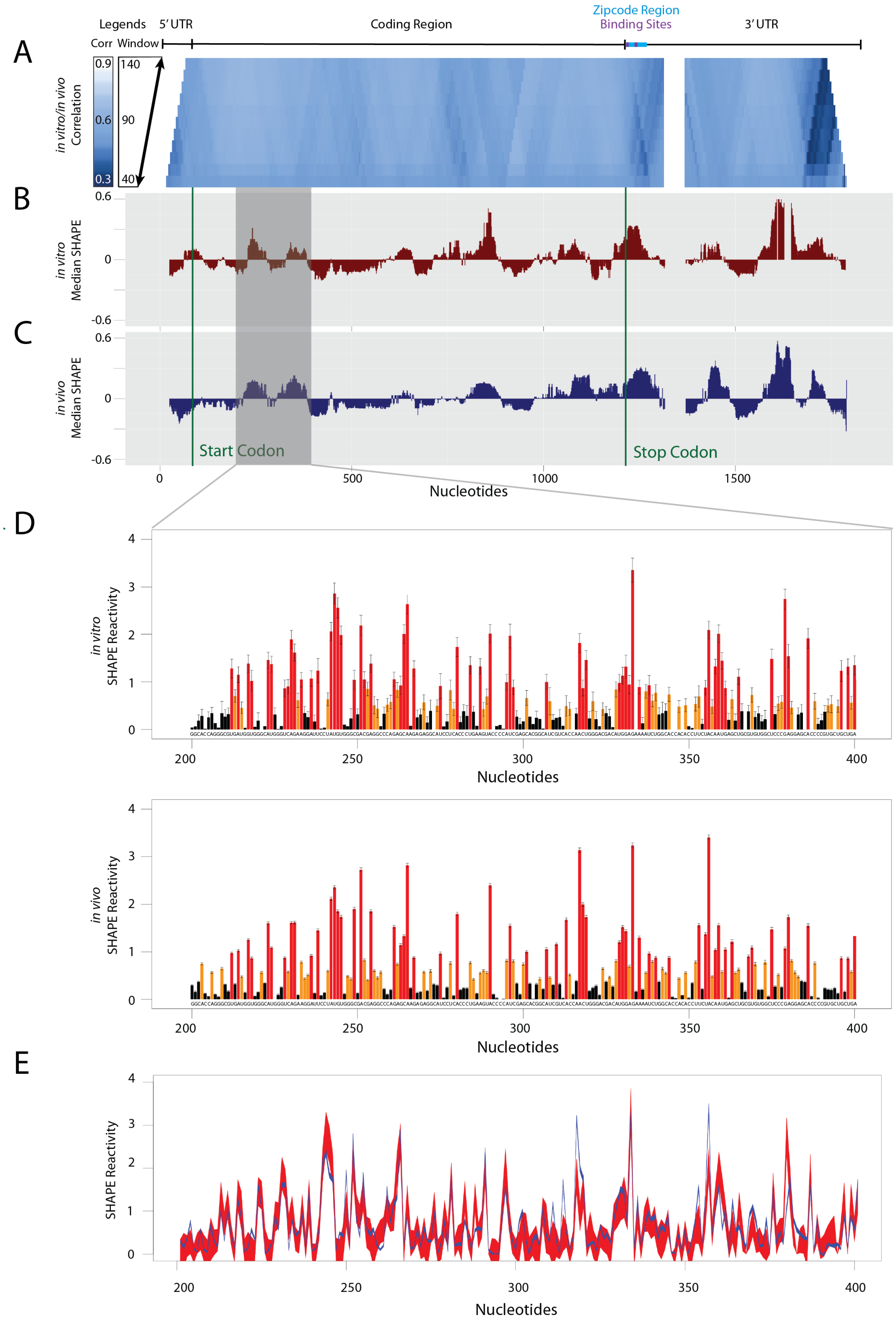

Figure S3. Comparison of similar *in vitro* and *in vivo* structure for the human *β-actin* mRNA. A) We calculated the Pearson correlation in windows between the SHAPE reactivities collected *in vitro* and *in vivo* for the *β-actin* mRNA. For each step of the trapezoid from bottom to top, the window size increases by five nucleotides from 40 to 140. High correlation (white) corresponds to areas that are similar in structure and low correlation (blue) corresponds to areas

that are different in structure. The distances from the median SHAPE value for B) *in vitro* and C) *in vivo β-actin* were calculated in 50 nucleotide windows. Segments with reactivities above the median are less structured than segments with reactivities below the median. The gray panel highlights a region in which the SHAPE reactivity is the same *in vitro* and *in vivo*. D) This similarity is reflected in the *in vitro* (top) and *in vivo* (bottom) SHAPE traces. E) The *in vitro* (red) and *in vivo* (blue) SHAPE traces were overlaid for this region. The thickness of the line corresponds to the error. Structure probing was performed using the high throughput SHAPE-MaP technique. The zipcode region (bright blue) and two zipcode protein-binding sites (purple) are labeled above the windowed correlation and at the bottom of the SHAPE traces.

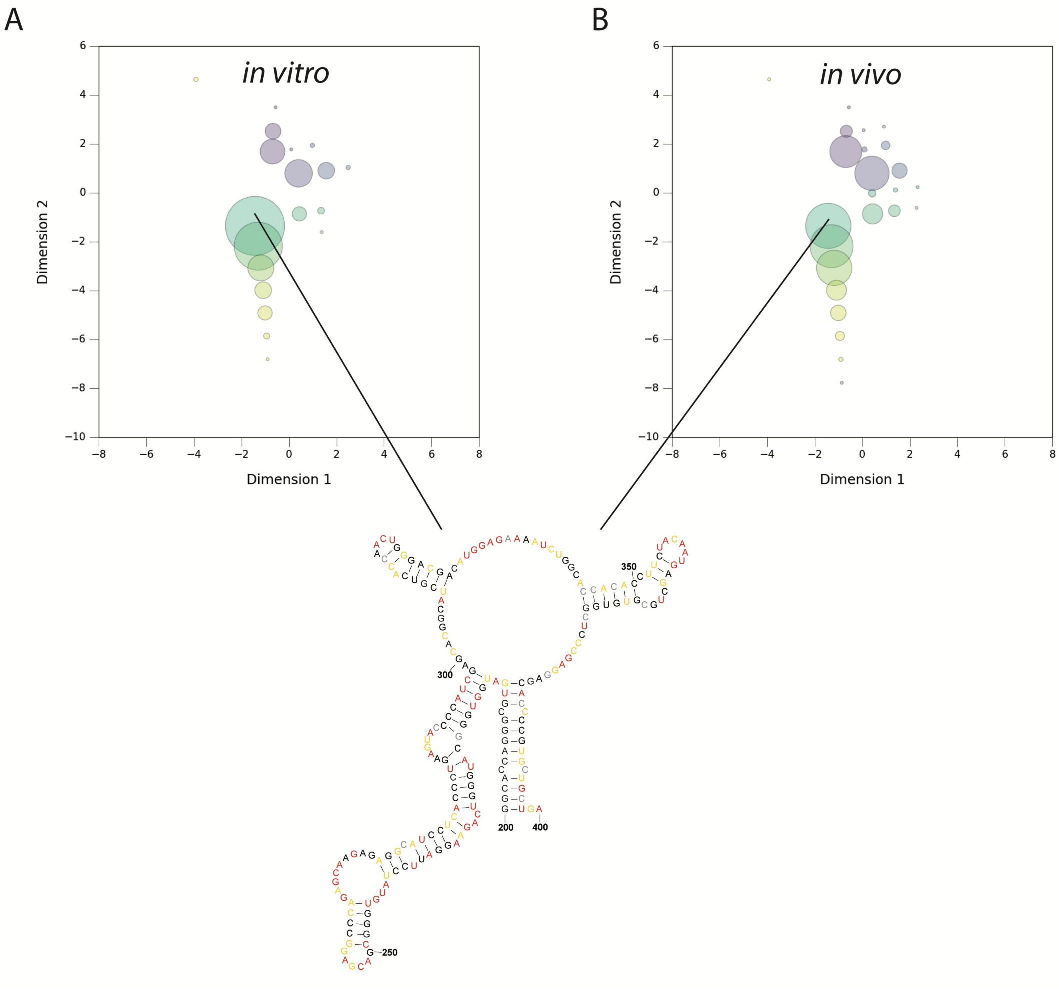

Figure S4. Similar *in vitro* and *in vivo* ensembles for human *β-actin* mRNA. Generation of structures for the *β-actin* mRNA ensemble was guided by the *in vitro* and *in vivo* SHAPE data. The 200-nucleotide regions were folded separately. We compared the visualizations for A) *in vitro* and B) *in vivo* SHAPE-guided ensembles for a region where SHAPE reactivities were expected to be the same. The visualization confirms that the *in vitro* and *in vivo* ensembles are the same. The medoid structure for the most common cluster is shown (center).

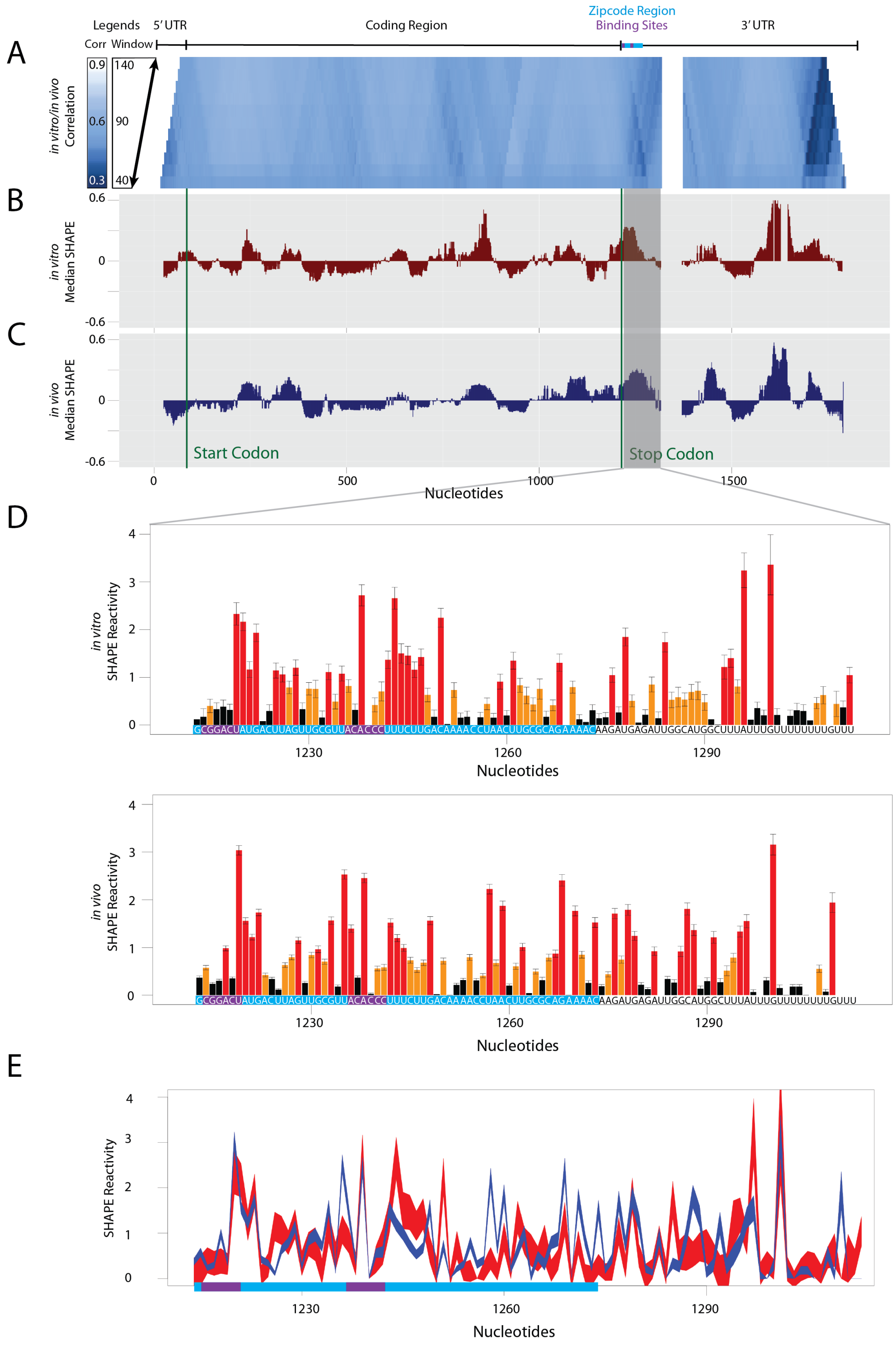

Figure S5. Comparison of different *in vitro* and *in vivo* structure for the human *β-actin* mRNA. A) We calculated the Pearson correlation in windows between the SHAPE reactivities collected *in vitro* and *in vivo* for the *β-actin* mRNA. For each step of the trapezoid from bottom to top, the window size increases by five nucleotides from 40 to 140. High correlation (white)

corresponds to areas that are similar in structure and low correlation (blue) corresponds to areas that are different in structure. The distances from the median SHAPE value for B) *in vitro* and C) *in vivo β-actin* were calculated in 50 nucleotide windows. Segments with reactivities above the median are less structured than segments with reactivities below the median. The gray panel highlights a region in which the SHAPE reactivity is different *in vitro* and *in vivo*. D) This difference is reflected in the *in vitro* (top) and *in vivo* (bottom) SHAPE traces. E) The *in vitro* (red) and *in vivo* (blue) SHAPE traces were overlaid for this region. The thickness of the line corresponds to the error. Structure probing was conducted using the high throughput SHAPE-MaP technique. The zipcode region (bright blue) and two zipcode protein binding sites (purple) are labeled above the windowed correlation and at the bottom of the SHAPE traces.

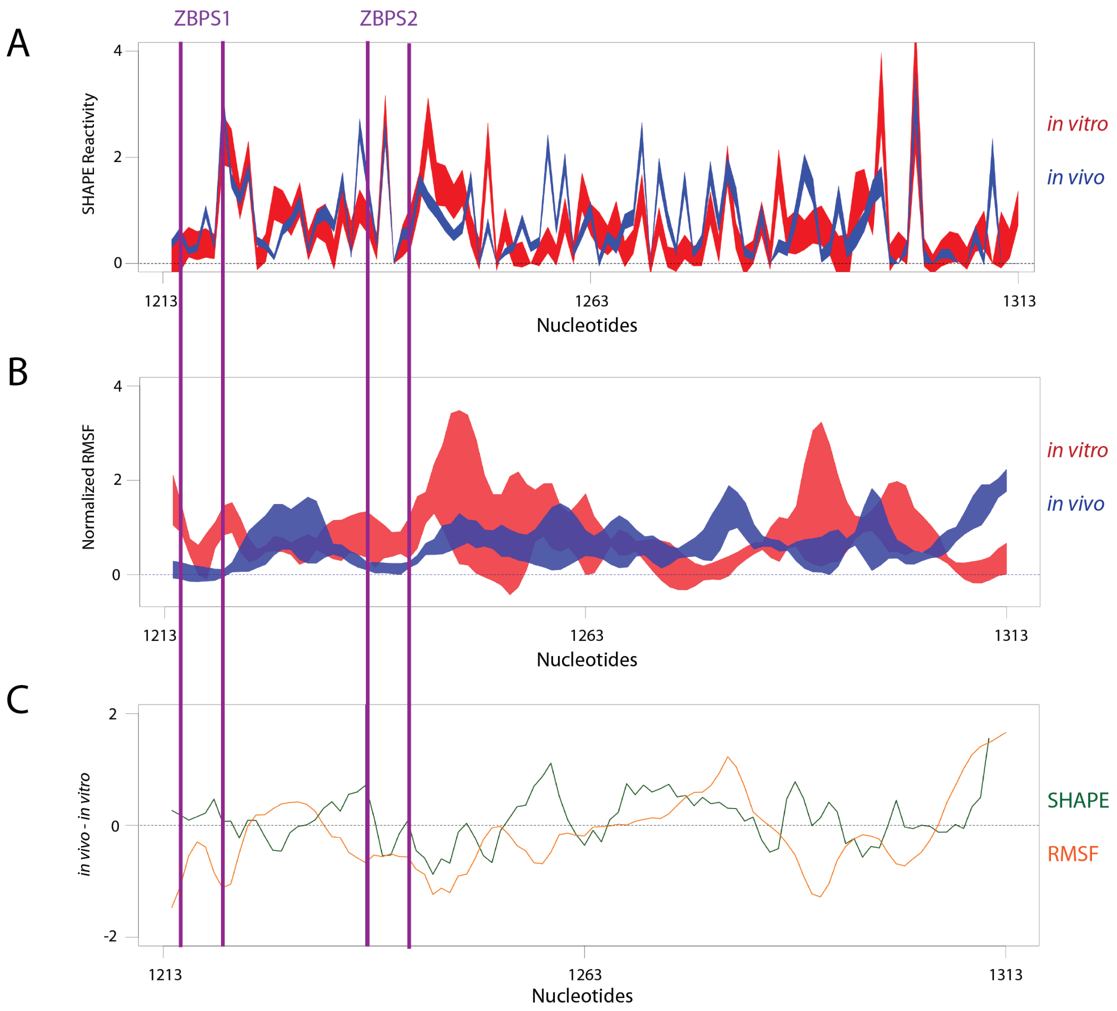

Figure S6. Comparison of different *in vitro* and *in vivo* flexibility for the human *β-actin* mRNA. A) The *in vitro* (red) and *in vivo* (blue) SHAPE traces were overlaid for the zipcode region of *β*-actin. The thickness of the line corresponds to the error. Structure probing was performed using the high throughput SHAPE-MaP technique. B) The normalized Root Mean Square Fluctuation (RMSF) for *in vitro* (red) and *in vivo* (blue) *β*-actin. The fluctuation is calculated from the 3D structural models shown in Figure 5. RMSF values were averaged over a 3-nucleotide moving window. Line thickness corresponds to standard error over three molecular dynamics simulations for each scenario. C) Comparison of the difference between in vivo and in vitro for SHAPE (green) and RMSF (orange). Values above zero indicate higher reactivities or RMSF for the *in vivo* sample. These values were averaged over a 3-nucleotide moving window. Values below zero indicate higher reactivities or RMSF for the *in vitro* sample. The zipcode binding sites are labeled with purple vertical lines for Figures A-C.

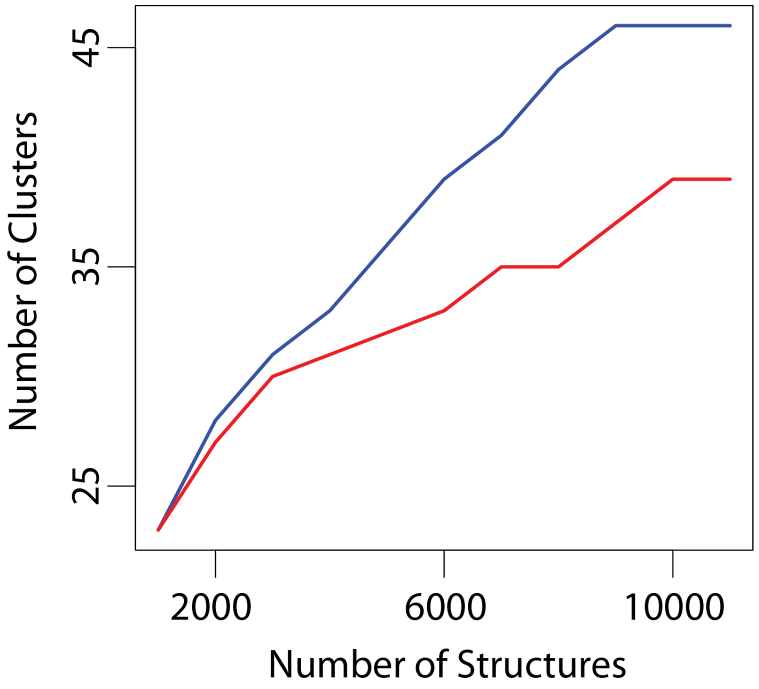

Figure S7. Comparison between single sequence stochastic sampling and single point mutant approach proposed in this manuscript for building a map of conformational space. In this analysis of the 200 nucleotide region of the *β-actin* mRNA shown in Figure S3, we compute the number of clusters as a function of sampled structures for the single point mutant strategy (blue) and single sequence sampling strategies (red). For the same total number of structures, we converge on just 39 structure clusters (red), and 46 using the single point mutant strategy (blue). Thus our strategy generally appears to create a more diverse space that converges on additional structures for ensemble visualization.

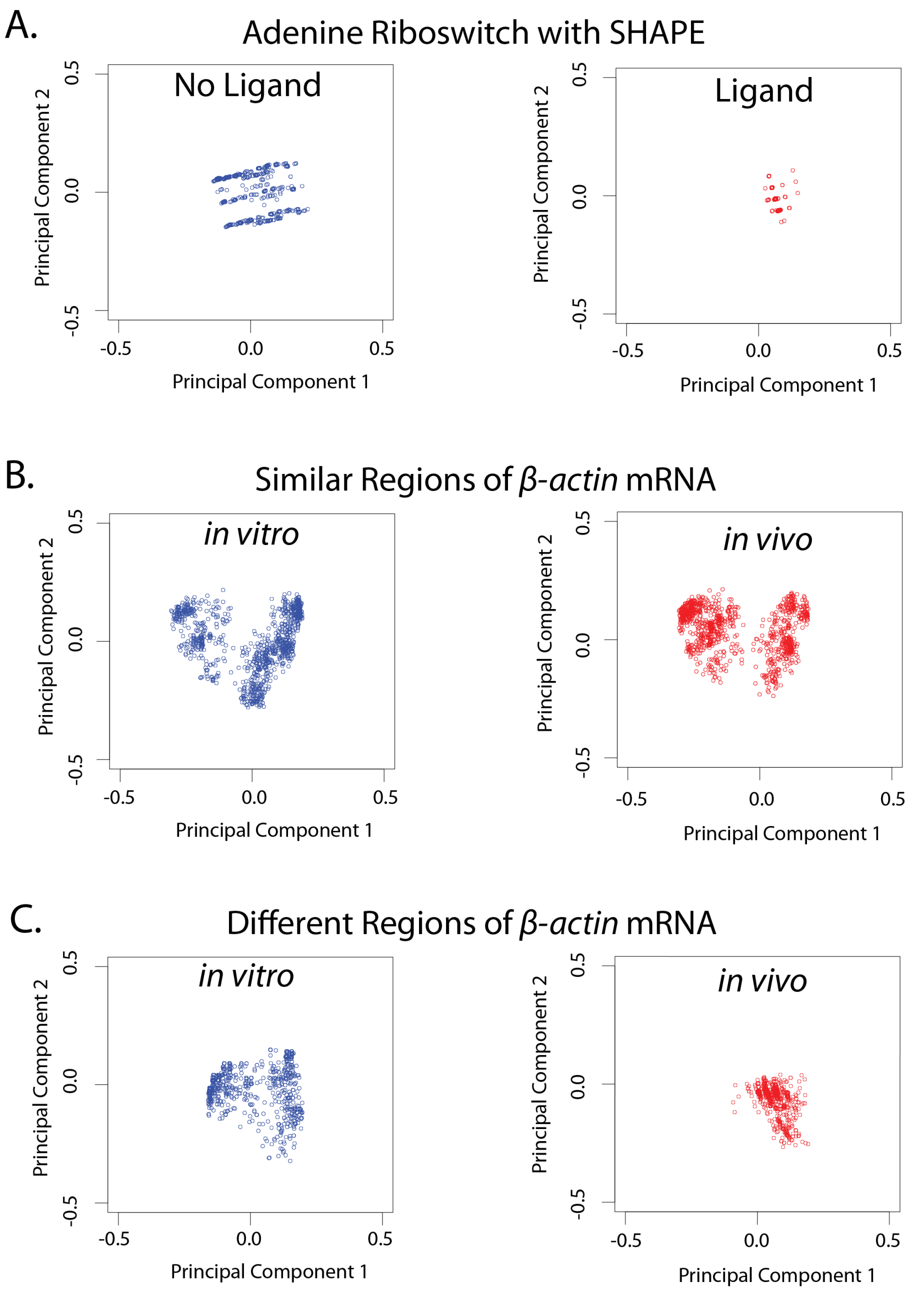

Figure S8. Projection of structural ensembles using principal components analysis (PCA). Representing RNA structure as a binary representation with 0 for unpaired positions and 1 for paired positions, we projected the ensemble onto the first two principal components from PCA (left/blue). Using the same eigenspace, we projected the comparison structural ensemble (right/red). A) We visualize the wild type adenine riboswitch ensemble with SHAPE from Figure 3B. Although the ensembles look different, the shift from "off" conformations to "on" conformations is not obvious in this visualization. B) We projected the region of the *β-actin* mRNA from Figure 4 where we expected the ensembles to be different *in vitro* and *in vivo* based on the Pearson correlation coefficients on the SHAPE-MaP data. The *in vivo* ensemble appears to

be shifting away from the preferred structure *in* vitro, but it is difficult to see the magnitude of the shift based on this visualization. C) Using PCA, we visualized the region of the *β-actin* mRNA from Figure S3 where we expect the ensembles to be the same. The visualization captures the similarity for these ensembles. However, it is difficult to compare the diversity of the ensembles in this region (more diversity of structures) with the ensemble from the region shown in C (less diversity of structures).

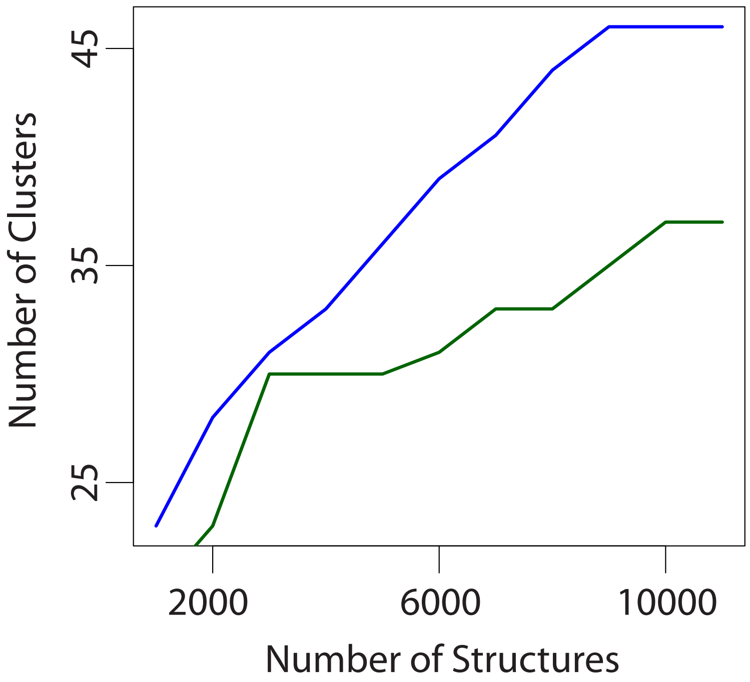

Figure S9. Comparison between hierarchical clustering with Euclidean distance as proposed in this manuscript (blue) and Jenson Shannon divergence (green) for building a map of conformational space. In this analysis of the 200 nucleotide region of the *β-actin* mRNA shown in Figure S3, we compute the number of clusters as a function of the number of mutants chosen from hierarchical clustering with 1000 structures for each mutant. The distances used in hierarchical clustering were the Euclidean distance (blue) and Jensen Shannon divergence (green). For the same total number of mutants, we converge on just 37 structure clusters (green), and 46 using the Euclidean distance strategy (blue). Thus our strategy generally appears to create a more diverse space that converges on additional structures for ensemble visualization that is the goal in this implementation of the visualization. Nonetheless, if users desire a simpler space for visualization, altering the distance metric used at this stage of the approach could be an interesting approach.

Documentation S1.

EnsembleRNA

September 20, 2016

**Title**	Visualize the structural ensemble for a given RNA
**Version**	1.0.0
**Author**	Chanin Tolson Woods
**URL**	http://ribosnitch-ensemblerna.rhcloud.com
**URL2**	http://ribosnitch.bio.unc.edu/software

Description

EnsembleRNA is a package for the visualization and comparison of RNA structural ensembles. This package creates a stable map of conformational space for a given RNA and its mutants. The map explores the most diverse conformational space and generates the structures using established Boltzmann-weighted suboptimal sampling algorithms. Using vector representation based on arc diagram nested loop patterns, EnsembleRNA projects clusters of structures from the map into two dimensions using metric multidimensional scaling. Individual RNA ensembles are visualized in this space by varying the size of the structure clusters in a bubble plot.

The sequence from the FASTA file is the reference used to create the map of conformational space, unless otherwise specified. To compare two reference structures, the same sequence or set of structures must be used to create the map of conformational space. Larger RNAs may require more sequences for a stable visualization. Selective 2'-hydroxyl acylation and primer extension (SHAPE) data can be included to guide the prediction of the reference ensemble.

Note: EnsembleRNA is written for use on a Linux/Unix-type operating system. Use of EnsembleRNA in any instance requires the installation of the numpy, jinja2, ipython, mpld3, matplotlib, scipy, and sklearn modules for Python. Also required is the RNAstructure package from the Mathews lab.

**Depends**	Python 2.7 or Python 3.5
**License**	GPL (>=3)
**Imports**	numpy, jinja2, ipython, mpld3, matplotlib, and sklearn
**Required**	RNAstructure

Table of Contents

Imports and Requirements 3

Installation 3

Usage 4

Options 4

Output 5

Troubleshooting 5

- Diagonal line visualization 5
- Selecting medoid structure 6
- Single point visualization 5
- Outer loop visualization 7
- Increasing map coverage 8

Documentation References 9

Imports and Requirements

For use on a Linux/Unix operating system

1) python (recommended version 2.7 or 3.5)

2) numpy (recommended 1.11.0)
   pip install numpy

3) scipy (recommended version 0.17.1)
   pip install scipy

4) sklearn (recommended version 0.0)
   pip install sklearn

5) jinja2 (recommended version 2.8)
   pip install jinja2

6) ipython (recommended version 4.2.0)
   pip install ipython

7) mpld3 (recommended version 0.2)
   pip install mpld3

8) matplotlib (recommended version 1.5.1)
   pip install matplotlib

9) RNAstructure (recommended version 5.8)
   http://rna.urmc.rochester.edu/RNAstructureWeb/
   Download command-line applications for your platform
   Extract to /usr/local/bin (or directory of your choice)
   Add following 2 lines to ~/.bash_profile (path may be different)
      export PATH=$PATH:/usr/local/bin/RNAstructure/exe
      export DATAPATH=/usr/local/bin/RNAstructure/data_tables

Installation

1) Download requirements listed above

2) Download EnsembleRNA package

3) Place package in /usr/local/bin (or directory of your choice)

4) tar -zxvf ensemblerna (extract)

5) cd ensemblerna (enter extracted directory)

6) sudo python setup.py install (install ensemblerna as python module)

7) ensemblerna -h (test installation in any directory)

Usage

ensemblerna <fasta file> <output directory> [options]

Options

General

-h, --help	show this help message and exit
-v, --version	show program's version number and exit

Inputs

-sh --shape	Includes shape data in the reference ensemble prediction. Ignored if -d flag is used (Default is None)
-d --db	Dot-bracket structures for reference ensemble (Default is None)
-m --map	Sequence to create the map of conformational space. Ignored if -md flag is used (Default is reference fasta file)
-md --mapdb	Dot-bracket structures for the map of conformational space. A previously created map can be used to project new ensembles onto the same space (Default is None)
-s --size	Number of mutants for the map of conformational space. Higher numbers increase structural diversity. Ignored if –md flag is used (Default is 10)
-p --plotmap	Plot the map T/F (Default is T)
-r --range	Range of nucleotides to visualize. Predicted structures will include the full length of the input RNA but only the given range will be plotted (Default is 1 to sequence length)
-pi --plotinteractive	Plot the interactive file T/F (Default is T)
-th --threadmax	Maximum number of threads for multi-threading. (Default is 1)
-i --ignorestems	Ignore stems with fewer than i base pairs. (Default is 3)
-n --num	Number of Boltzmann sampled structures to produce for the visualization (Default is 1000)

RNAstructure

-maxd --maxdistance	The maximum number of bases between the two nucleotides in a pair (Default is no restriction)
-t --temperature	Temperature at which the calculation takes place in Kelvin (Default is 310.15 K)
-si --SHAPEintercept	The intercept used with SHAPE restraints. Ignored if -d flag is used (Default is -0.6 kcal/mol)
-sm --SHAPEslope	The slope used with SHAPE restraints. Ignored if -d flag is used. (Default is 1.8 kcal/mol)

Output

For both reference and map of conformational space
.csv CSV file with cluster number, cluster size, and representative structure
.db Dot-Bracket file with structures
.pdf PDF file with visualization plot
.png PNG file with visualization plot

Interactive visualization
.html HTML file with interactive plotting

Troubleshooting

Diagonal line visualization

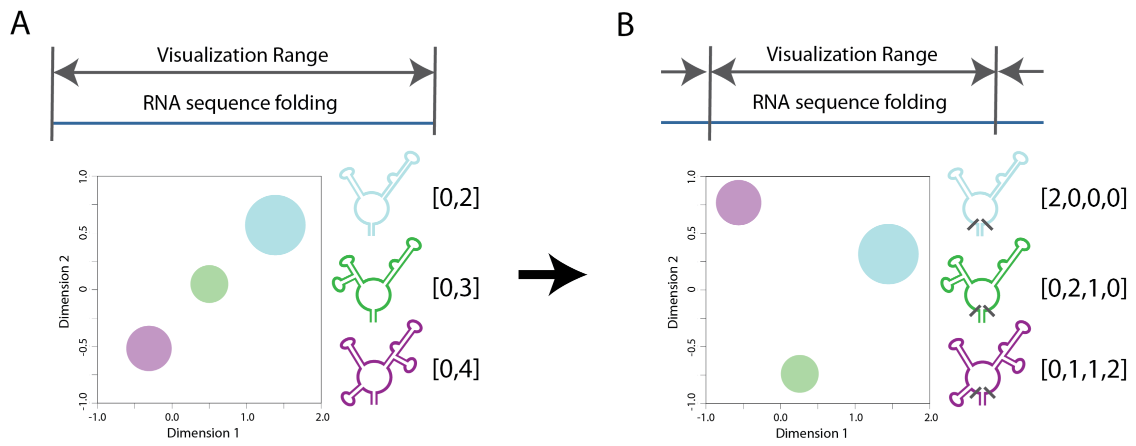

Problem If all bubbles lie on a diagonal line, there is correlation between dimensions 1 and 2 (A). By default, EnsembleRNA defines RNA structure based on the outermost stacks and loops (the most abstracted representation). In this case, the structures are similar from the level of the outermost stack, but interesting differences may exist for loops within that outer stack.

Solution To address this problem, the full-length of the RNA can be folded, while the visualization is focused on a shorter range that excludes the outer stack (B). This change reveals the more subtle differences between structures.

Example For a 250 nucleotide RNA, include the entire sequence in the fasta file. Only visualize the range from nucleotide 50 to 200 using the range flag (-r or --range).

ensemblerna <fasta file> <output directory> **-r 50 200**

Selecting medoid structure

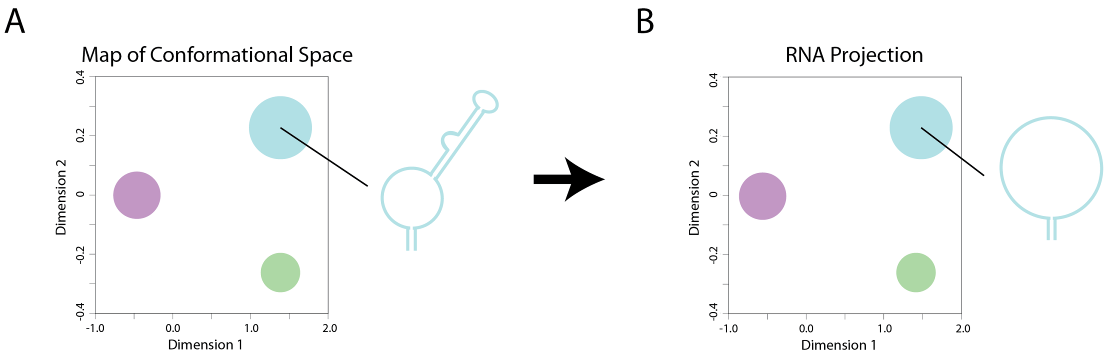

Problem The default medoid structure is chosen from the map of conformational space (A). Using this medoid keeps the representation consistent between different ensembles projected onto the same space. However, the best representative structure for the projected RNA may be different.

Solution A structure selected from the projected RNA ensemble may be more representative (B). Alternatively, the minimum free energy structure from either the map or the projected RNA ensemble can be used.

Example Check the .db file in the output folder.

Single point visualization

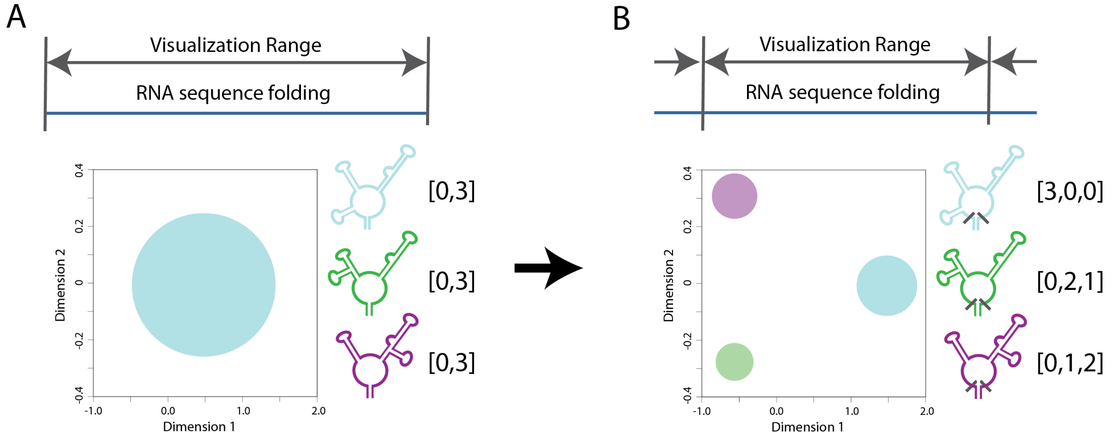

Problem If all structures are placed in a single cluster, the visualization may be too broad (A). By default, EnsembleRNA defines RNA structure based on the outermost stacks and loops (the most abstracted representation). In this case, the structures are the exact same from the level of the outermost stack, but interesting differences may exist for loops within that outer stack.

Solution To address this problem, the full-length of the RNA can be folded, while the visualization is focused on a shorter range that excludes the outer stack (B). This change reveals the more subtle differences between structures.

Example For a 250 nucleotide RNA, include the entire sequence in the fasta file. Only visualize the range from nucleotide 50 to 200 using the range flag (-r or --range).

ensemblerna <fasta file> <output directory> **-r 50 200**

Outer loop visualization

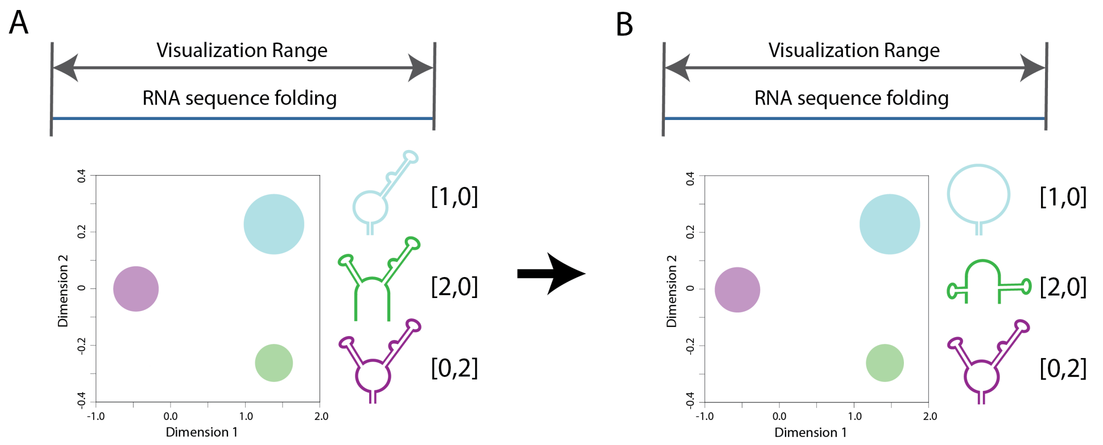

Problem RNA clusters with only outer loops are considered to be more similar to each other than those clusters with an outer stack (A). The clusters with only outer loops are most often very diverse groupings. While some structures may be similar to those with outer stacks, many structures will be quite different.

Solution Our nestedness representation method accounts for this increased diversity in clusters with only outer loops (B). Looking at the cluster medoid may be useful in assessing the similarity of these clusters to those with outer stacks.

Example Check the .csv file or the .html file in the output folder.

Increasing map coverage

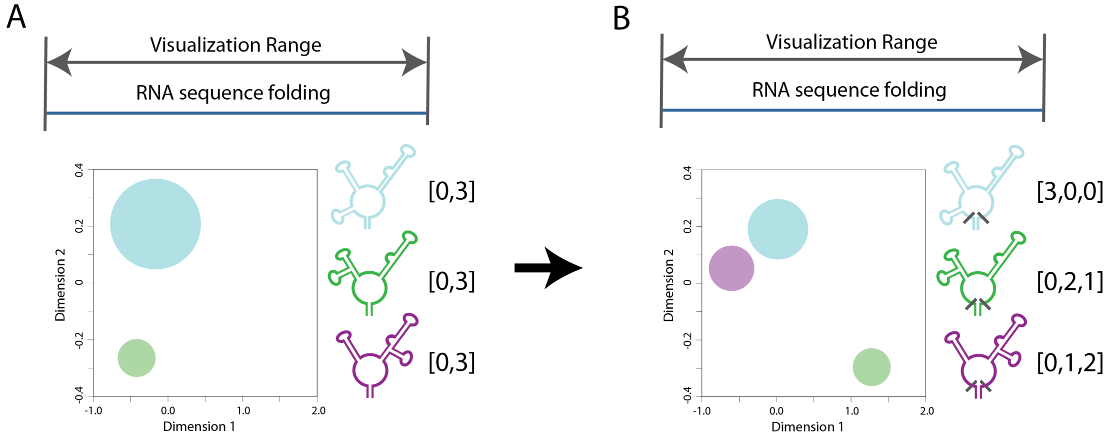

Problem The default map size (the number of single point mutants included in the map) is automatically set to 10. This is a reasonably size for RNAs of shorter length (100-200 nucleotides). However, longer RNAs will likely require larger map sizes to sufficiently explore the structural space for an RNA.

Solution Increase the map size in increments until the number of clusters structures converges. At this map size, additional single point mutants will not increase the structural diversity in the map of conformational space. The optimal map size varies by RNA.

Example For an 800 nucleotide RNA, include the entire sequence in the fasta file. Increase the map size from 10 to 100.

ensemblerna <fasta file> <output directory> **-s 100**

Documentation References

Suboptimally sampled structures are generated using the RNAstructure package
http://rna.urmc.rochester.edu/RNAstructureWeb/
Version 5.8 (references 2, 3, 4, and 5)

1. D.H. Mathews. 2004. Using an RNA Secondary Structure Partition Function to Determine Confidence in Base Pairs Predicted by Free Energy Minimization. RNA, 10:1178-1190. (2004).
2. S. Duan, D.H. Mathews, D. H. Turner. 2006. Interpreting Oligonucleotide Microarray Data to Determine RNA Secondary Structure. Application to the 3' End of Bombyx mori R2 DNA. Biochemistry, 45:9819-9832.
3. D.H. Mathews. 2006. Revolutions in RNA Secondary Structure Prediction. Journal of Molecular Biology, 359:526-532.
4. S. Wuchty, W. Fontana, I. L. Hofacker, P. Schuster. 1999. Complete Suboptimal Folding of RNA and the Stability of Secondary Structures. Biopolymers, 49:145-165.
5. Y. Ding and C.E. Lawrence. 2003. A Statistical Sampling Algorithm for RNA Secondary Structure Prediction. Acids Research, 31:7280-7301.
6. E.J. Merino, K. A. Wilkinson, J. L. Coughlan and K. M. Weeks. 2005. RNA structure analysis at single nucleotide resolution by selective 2-hydroxyl acylation and primer extension (shape). J Am Chem Soc, 127:4223-4231.
7. K.E. Deigan, T. W. Li, D. H. Mathews and K. M. Weeks. 2009. Accurate SHAPE-directed RNA Structure Determination. Proceedings of the National Academy of Sciences USA, 106:97-102.
8. K. Pearson. 1901. On Lines and Planes of Closest Fit to Systems of Points in Space. Philosophical Magazine, 11:559–572.
9. D.B. Carr, R. J. Littlefield, W.L. Nicholson, J.S. Littlefield. 1987. Scatterplot Matrix Techniques for large N. Journal of the American Statistical Association, 389:424-436.
10. J. Ritz, J. Martin and A. Laederach. 2012. Evaluating our ability to predict the structural disruption of RNA by SNPs. BMC Genomics, 13(Suppl 4):S6.

Supporting References

1. P. Steffan, V. Bjorn, M. Rehmsmeier, J. Reeder and R. Giegerich. 2006. RNAshapes: an integrated RNA analysis package based on abstract shapes. 22:500-503.
2. Y. Ding and C. Lawrence. 2003. A statistical sampling algorithm for RNA secondary structure prediction. 31:7280-7301.
3. D. H. Matthews, M. D. Disney, J. L. Childs, S. J. Schroeder, M. Zuker and D. H. Turner. 2004. Incorporating Chemical Modification Constraints into a Dynamic Programming Algorithm for Prediction of RNA Secondary Structure. 101:7287-7293.
4. N. A. Siegfried, S. Busan, G. M. Rice, J. A. Nelson and K. M. Weeks. 2014. RNA motif discovery by SHAPE and mutational profiling (SHAPE-MaP). 11:959-965.
